# Mapping the biogenesis of forward programmed megakaryocytes from induced pluripotent stem cells

**DOI:** 10.1126/sciadv.abj8618

**Published:** 2022-02-16

**Authors:** Moyra Lawrence, Arash Shahsavari, Susanne Bornelöv, Thomas Moreau, Rebecca McDonald, Thomas M. Vallance, Katarzyna Kania, Maike Paramor, James Baye, Marion Perrin, Maike Steindel, Paula Jimenez-Gomez, Christopher Penfold, Irina Mohorianu, Cedric Ghevaert

**Affiliations:** 1 Cambridge Stem Cell Institute, Jeffrey Cheah Biomedical Centre, Cambridge Biomedical Campus, Puddicombe Way, Cambridge CB2 0AW, UK.; 2 Department of Haematology and NHS Blood and Transplant, University of Cambridge, Cambridge, UK.; 3 Bit Bio, Discovery Drive, Cambridge Biomedical Campus, Cambridge CB2 0AX, UK.; 4 Cancer Research UK Cambridge Institute, University of Cambridge, Li Ka Shing Centre, Robinson Way, Cambridge CB2 0RE, UK.; 5 Department of Physiology, Development and Neuroscience, University of Cambridge, Downing Street, Cambridge CB2 3EG, UK.

## Abstract

Platelet deficiency, known as thrombocytopenia, can cause hemorrhage and is treated with platelet transfusions. We developed a system for the production of platelet precursor cells, megakaryocytes, from pluripotent stem cells. These cultures can be maintained for >100 days, implying culture renewal by megakaryocyte progenitors (MKPs). However, it is unclear whether the MKP state in vitro mirrors the state in vivo, and MKPs cannot be purified using conventional surface markers. We performed single-cell RNA sequencing throughout in vitro differentiation and mapped each state to its equivalent in vivo. This enabled the identification of five surface markers that reproducibly purify MKPs, allowing us insight into their transcriptional and epigenetic profiles. Last, we performed culture optimization, increasing MKP production. Together, this study has mapped parallels between the MKP states in vivo and in vitro and allowed the purification of MKPs, accelerating the progress of in vitro–derived transfusion products toward the clinic.

## Teaser

A surface marker panel can purify megakaryocyte progenitor cells from mixed cultures, allowing their production to be optimized.

## INTRODUCTION

Platelets are the cells responsible for clotting. Every year in the United Kingdom, 280,000 platelet units are transfused into patients with a low platelet count, known as thrombocytopenia, to prevent hemorrhage. Most of the patients receive platelet transfusions either after hemorrhage or bone marrow insufficiency following cancer treatment or hematopoietic malignancy (*1*). Platelet units are collected from donors and matched for ABO blood type and Rhesus D (*1*). However, previous transfusion recipients and multiparous women may become immunized against foreign major histocompatibility complex (MHC) class I or other antigens on the platelet surface, adding even more complexity to the matching process (*2*). In addition, short platelet shelf life of 5 to 7 days and restricted donor availability mean that platelets have the most precarious supply chain of all blood components, and shortages may occur.

**Fig. 1.**
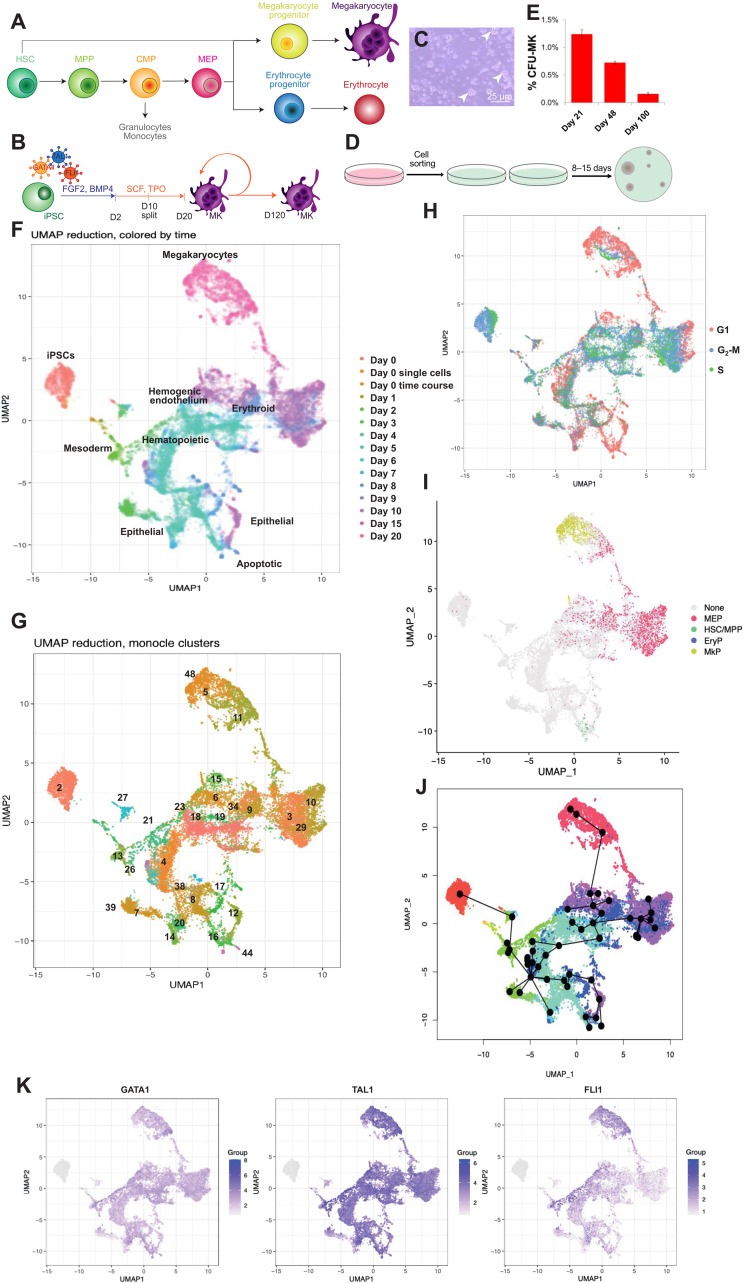
Single-cell sequencing of MK differentiation from iPSCs allows the comparison to human in vivo hematopoiesis and trajectory analysis. (**A**) Overview of hematopoiesis in vivo. (**B**) Schematic of protocol (*17*). iPSCs are lentivirally transduced with *GATA1*, *TAL1*, and *FLI1*. After 20 days, mature megakaryocytes (MKs) are formed, which can persist for up to 120 days. (**C**) Bright-field image of A1ATD1 MKs on D20 of differentiation. Arrowheads, proliferative clusters. Scale bar, 25 μm. (**D**) Schematic of colony-forming unit (CFU) assay. MKs are sorted into semisolid methylcellulose containing SCF and TPO. After 9 to 15 days, emergent colonies can be scored. (**E**) Percentage of colony formation in an A1ATD1 culture in CFU assays at D21, D48, and D100 of culture. (**F**) UMAP embedding of scRNA-seq of MK differentiation time course (10x Genomics). Legend: time point during differentiation. (**G**) Monocle clusters on time-course data; 51 clusters identified using density peak–based clustering. Clusters mentioned in the section “Mapping MK differentiation in vitro using scRNA-seq” are labeled. Full plot in fig. S3A. (**H**) UMAP of cell cycle stage, inferred from the expression of cell cycle–associated transcripts. (**I**) Random forest-predicted cell types on single-cell time-course data. Cell types learned on 10x Genomics HSPCs from human bone marrow, spleen, and peripheral blood. MkP, megakaryocyte progenitor; EryP, erythroid progenitor. (H) Donor 1, 1% D0 cutoff, threshold of 0.53. (**J**) Slingshot trajectory analysis of clusters from (G) colored by time point. (**K**) UMAP of un-normalized *GATA1*, *TAL1*, and *FLI1* expression from lentiviral transgenes.

Megakaryocytes (MKs) are large, multinucleated polyploid cells (*3*) that differentiate from hematopoietic stem cells (HSCs) in the bone marrow. MKs make up 0.01 to 0.03% of nucleated bone marrow cells (*4*), and collectively, they are estimated to produce 1 × 10^11^ to 2 × 10^11^ platelets daily (*5*). The long-standing model of HSC differentiation is a hierarchical model (Fig. 1A) where HSCs differentiate to form multipotent progenitors (MPPs), which undergo transit-amplifying divisions and can generate all mature blood cells (*6*). As these differentiate and become more lineage restricted along the path to MK differentiation, common myeloid progenitors (CMPs) are formed (*7*) that subsequently differentiate to become MK erythroid progenitors (MEPs), which are restricted to MK and erythrocyte production (*8*). MK progenitors (MKPs) differentiate from MEPs and can only form MKs (*8*, *9*). These were first observed in the human MEPs produced from mobilized peripheral blood–derived CD34^+^ cells (*9*). MKPs retain very little capacity to produce erythroid cells and almost exclusively produce MKs in colony-forming assays (*9*). They express MK-associated proteins such as CD41, CD61, VWF, CLU, and NF1B with CD42 expression heralding the extinction of erythroid colony-forming potential (*9*).

There is also evidence to suggest that MKs can differentiate directly from earlier hematopoietic intermediates. Distinct subsets of HSCs/MPPs express MK markers while still in the stem cell compartment, from which MKs may be able to differentiate directly in times of stress (*10*). MKPs can also emerge directly from CMPs without becoming MEPs (*11*).

After the discovery of induced pluripotent stem cells (iPSCs) (*12*), they were rapidly adopted as a source for in vitro platelet generation as they were easily expandable unlike HSCs. Protocols for MK production from iPSCs can be divided into two main groups: one relying on sequential cytokine cocktails to guide PSCs through development, termed as directed differentiation (*13*–*16*), and the other involving the overexpression of key transcription factors, termed as forward programming (*17*, *18*). The latter tended to produce higher-purity MKs and use minimal cytokines. Some groups also produced immortalized MKPs, which made cultures easily scalable but may have safety drawbacks due to genetic instability and a higher tumorigenicity risk to recipients (*19*). iPSCs can be genetically modified, promoting efficient differentiation using inserted cassettes for the overexpression of differentiation-promoting factors (*20*, *21*) and allowing MHC class I deletion to reduce the immunogenicity of manufactured platelets (*13*, *19*).

We previously published a protocol for mature MK differentiation from iPSCs using two cytokine combinations (*17*) through the overexpression of *GATA1*, *TAL1*, and *FLI1*. These MKs produce functional platelets that can contribute to thrombus formation (*17*). Early time points of the culture produce both erythroid cells and MKs, tuned by the rheostat of *FLI1* expression (*22*), mirroring in vivo megakaryopoiesis (*23*). One of the key characteristics of the cultures is their ability, beyond reaching maturity at day 20 (D20), to survive for a further 100 days while maintaining cell proliferation and purity by CD41 and CD42 expression (*17*), indicating the presence of cells capable of reconstituting the culture when mature MKs fragment to generate platelets. We hypothesized that the culture is maintained by highly proliferative MKPs, mirroring in vivo MKPs.

Here, we used single-cell RNA sequencing (scRNA-seq) to map differentiation from iPSCs to mature MKs in vitro. We investigated whether the progenitors generated during in vitro MK differentiation mirrored hematopoietic progenitors in vivo and found that only the more mature MKs and erythroid cells had a transcriptional signature similar to human primary MEPs and MKPs. Using a combination of scRNA-seq and machine learning approaches on index sort data, we identified a combination of five previously unidentified surface markers that reproducibly isolated and purified MKPs from MK cultures derived from both iPSCs and peripheral blood CD34^+^ HSCs. MKPs purified using this approach provided new insights into MKPs’ transcriptional and epigenetic state. Last, we optimized culture conditions for the expansion of MKPs, showing that this increased culture efficiency and postponed long-term culture exhaustion, potentially facilitating subsequent large-scale manufacturing of a clinical product.

## RESULTS

### Forward programmed MK cultures from iPSCs contain MKPs

We previously described a robust and reproducible protocol for the production of mature, functional MKs from iPSCs, catalyzed by the lentiviral overexpression of *GATA1*, *TAL1*, and *FLI1* (Fig. 1B). The culture is adherent until D10 when it is disrupted and put into suspension. Mature MKs are harvested from D20. The cells acquire CD235 expression, then CD41 expression, and lastly CD42 expression as they become mature (*17*, *22*), and the cultures can produce a constant supply of mature MKs for up to 120 days. Proliferating cell clusters are often seen in these cultures (Fig. 1C), suggesting that they are maintained by dividing MKPs at the center of each cluster. Progenitors can be quantified in colony formation assays whereby a suspension of cells is seeded into semisolid medium containing cytokines. Each progenitor will form a “colony” that can be counted using microscopy and phenotyped by flow cytometry. Colony assays (Fig. 1D) performed on live-sorted MK cultures suggested that colony-forming MKPs make up only around 1% of early cultures and diminish as the culture ages (Fig. 1E). This confirmed that as the progenitors disappear, the cultures become exhausted. Morphologically, these progenitors are identical to the surrounding cells and cannot be identified using classical hematopoietic and/or MK surface markers (such as CD34, KIT, KDR, or CD61; fig. S1, A and B).

### Mapping MK differentiation in vitro using scRNA-seq

To understand the processes of MK differentiation in vitro, we fixed A1ATD1 cells for 10x Genomics scRNA-seq every 24 hours during the first 10 days of the MK differentiation protocol and also on D15 and D20 when the MKs are mature. We also designed a unique strategy for transgene fingerprinting (fig. S1C): The cells had been differentiated with the aid of three lentiviral transcription factors, stably inserted into the genome. Their downstream lentiviral sequences allowed the amplification of their transcripts from cDNA with specially designed primers that were substituted for the V(D)J primers during target enrichment. This allowed an overview of the gene expression of these progenitors and also the transgene quantities catalyzing their formation.

Quality control was performed as detailed in fig. S2 and Materials and Methods. All cells were projected on a Uniform Manifold Approximation and Projection (UMAP), colored by time point (Fig. 1F and fig. S2M). Fifty-one clusters were detected by Seurat, and we set Monocle to find the same number of clusters (Fig. 1G and fig. S3A). The set of cluster markers and enrichment terms (tables S3 and S4) were used to assess cluster identity and function.

iPSCs were located at the top left of the UMAP (Fig. 1F) and separated into three clusters (“iPSC” clusters 2, 21, and 27) of which the main cluster (cluster 2) had the highest potency, expressing higher levels of markers of the naïve (fig. S3B) and primed pluripotent states (fig. S3C). As cells left cluster 2, they lost the expression of pluripotency-associated markers and acquired a mesoderm identity, expressing *HAND1* (“mesoderm” clusters 4, 7, 8, 13, 26, 38, 39, and 43; fig. S3D). From here, the cells bifurcated onto two paths. The first progressed toward the bottom of the UMAP plot and expressed higher levels of mitochondrial transcripts. Some of these cells apoptosed at the bottom when the cells were dissociated from adhesive culture on D10, as seen by the enrichment of cluster 44 for apoptosis-associated gene ontology (GO) terms. Other cells expressed epithelial markers such as *KRT8*, *KRT18*, and *KRT19* (“epithelial” clusters 8, 12, 14, 16, 17, 44, 45, and 47; fig. S3D), indicating that they moved toward an epithelial fate. These cells were lost as the cells were dissociated on D10. A second branch of cells progressed toward the central part of the graph and expressed markers classically associated with hematopoiesis and hemogenic endothelium such as *CD34*, *KDR*, *CDH5*, and *PECAM1* (“hemogenic endothelium” clusters 4, 18, 19, 21, and 23; fig. S3D). *GATA1* and *TAL1* hematopoietic transcription factors were also expressed at this stage (fig. S3D). From D9, the cells transitioned toward an erythroid identity on the top right, characterized by *GYPA*, heme, and porphyrin synthesis and iron metabolism (“erythroid” clusters 3, 6, 9, 10, 15, 29, 33, 34, 37, 46, and 50). As these cells matured and moved toward the top of the graph, they acquired platelet-associated transcripts such as *ITGA2B* and lastly became mature MKs expressing *GP1BA* and high levels of *FLI1* (“megakaryocytes” clusters 5, 11, and 48) (fig. S3D).

We would expect MKPs to be found from an early time point in the culture as MK-producing cells are present before D10 (*22*). MKPs should also persist until the end of the time course as they support long-term culture. MKPs should therefore be found in clusters containing cells from multiple time points such as the hemogenic endothelium clusters or the erythroid clusters, both of which contained cells from D8 to the end of the time course.

The progenitors we observe in colony-forming assays are highly proliferative. Thus, we carried out cell cycle mapping of the differentiation process, partitioning cells into stages of the cell cycle according to their dominant transcripts. Consistent with reports that human pluripotent stem cells have a shortened G_1_ phase (*24*), the iPSCs on the top left had few cells in G_1_ (Fig. 1H). Correspondingly, as the iPSCs began to differentiate, they entered G_1_ phase, consistent with reports of G_1_ lengthening during differentiation (*25*). The erythroid cluster and the MK cluster segregated on the basis of cell cycle stage. Most of the MKs were in G_1_ phase, consistent with previous reports that G_1_-associated cyclins are required for the endomitosis involved in MK polyploidization (*26*). Differentiating cells also progressed to G_1_ phase as they neared the MK state toward the top right of the plot, indicating that one of these G_1_-dominant clusters may contain MKPs.

The cell classifications used in Fig. 1F were based on transcript enrichment relative to the rest of the dataset, which tells us that the MKs are, for example, more megakaryocytic than the rest of the dataset. It does not tell us, however, whether we generate cells that correspond to their in vivo counterparts. To investigate this, we compared our dataset to a dataset (*27*) containing 30,873 10x Genomics-sequenced CD19^−^CD34^+^ human hematopoietic stem and progenitor cells (HSPCs) from the bone marrow, peripheral blood, and spleen of two donors. A random forest model was trained on the cell types from the in vivo data and deployed to predict the cell type identities of the time series data (further details available in Materials and Methods). The results are shown in Fig. 1I (donor 1) and fig. S3E (donor 2). Very few primary HSCs or MPPs mapped to the iPSC differentiation dataset, and those that did were part of the apoptotic cluster (Fig. 1F). Although the cells found in the “hematopoietic” and “hemogenic endothelium” regions did express markers consistent with this identity such as *CD34*, *KDR*, *CDH5*, and *PECAM1*, we found that this was not the case for the expression of other markers defining HSCs (CD59^+^CD90/*THY1*^+^CD38^low/−^c-Kit^−/low^) and MPPs (CD90/*THY1*^−^ CD38^−^) (fig. S3D). None of the cells in these regions mapped to primary HSCs or MPPs (Fig. 1I and fig. S3E). Together, these data indicate that the forward programming of iPSCs to MKs is a process that specifies MK identity without passing through an HSC or HSC-like intermediate stage (table S5). This is consistent with our previous findings that in vitro MK differentiation did not generate cells capable of myeloid differentiation (*22*). From D9, when the cells began to express CD41, a marker of MEPs, we saw cells emerging that mapped to primary MEPs (Fig. 1I and fig. S3E), consistent with our previously published results demonstrating that D9 to D10 cells can undergo either MK or erythroid differentiation (*22*). Most cells generated after D9 mapped directly to primary MKPs (Fig. 1I and fig. S3E). On D9, the cells mapping to in vivo MKPs were the yellow points in cluster 6, making this cluster an attractive one for investigation as a potential MKP cluster (Fig. 1, G and I). We concluded from these data that forward programming enforces an accelerated patterning, which bypasses the usual hematopoietic hierarchy but generates end-stage progenitors faithfully recapitulating primary MKPs.

To map the path of the cells through differentiation, we performed trajectory analysis through the monocle clusters using Slingshot (*28*) (Fig. 1J and fig. S4A). The trajectory began with markers such as *EPCAM*, which is highly expressed in human pluripotent stem cells (*29*). The cells differentiated to mesoderm marked by transcripts such as *SMARCC*, which plays a key role in mesoderm formation during hematopoietic differentiation (*30*). Some of the cells became epithelial at the bottom of the graph, defined by transcripts associated with epithelial-to-mesenchymal transition such as *S100A4*. Other cells progressed toward early hematopoietic cells, propelled by key bone marrow niche–associated transcripts such as *RNPEP (**31**)* and cytokinesis factors such as *CLIC4* (*32*). Cells directly after this decision point (clusters 4, 20, and 21) were characterized by the expression of ribosomal transcripts, which also characterized the end of the trajectory toward mature MKs, indicating increased protein synthesis. A crucial part of the trajectory was seen in clusters 18 and 19, where the trajectory divided into MK and erythroid fates. Markers up-regulated here were also up-regulated in the MK clusters and the erythroid clusters. These cells can form both mature MKs and erythroid cells (*22*), demonstrating the functional relevance of the MEP state identified by trajectory analysis. The erythroid branch was defined by markers such as *TAL1*, which is associated with erythropoiesis (*16*). Last, the branch of the trajectory corresponding to mature MKs was defined by transcripts such as *PF4* and *GP9*, which are highly expressed in mature MKs (*33*, *34*). Trajectory analysis (Fig. 1J) allows us to pinpoint clusters 6, 15, and 34 (Fig. 1G) as clusters through which cells must pass to become mature MKs, implying the presence of MKPs in these clusters.

Next, we carried out targeted sequencing of lentiviral transcription factor–derived transcripts to investigate their contribution to specific trajectories (Fig. 1K and fig. S4B). *GATA1* and *TAL1* were highly expressed throughout differentiation to all cell fates; however, *FLI1* was expressed exclusively along the trajectory to mature MKs. Erythroid and epithelial clusters silenced *FLI1* expression, in agreement with previously published work that demonstrated that erythroid-committed MEPs silenced *FLI1* transgene expression, whereas MK-committed MEPs required *FLI1* expression to differentiate into MKs (*22*). Clusters 6, 15, and 34 expressed more *FLI1* than other clusters from the same time points, indicating that these clusters contained cells within the erythroid cluster that were destined to become MKs and could thus correspond to MKPs (Fig. 1K).

Together, our mapping allowed us to identify clusters 6, 15, and 34 (Fig. 1G) as particularly interesting and potentially containing MKPs. These clusters contained cells from D8 onward, as we would expect from an MKP cluster (Fig. 1F). These clusters expressed more *FLI1* than other cells from the same time points (Fig. 1K). Last, cells must pass through these clusters on their predicted trajectory toward the MK fate (Fig. 1J). We therefore hypothesized that this region corresponded to MKPs.

### Identifying MKP markers using a single-cell approach

We hypothesized that MKPs have a more proliferative transcriptional signature than the surrounding cultures and must be found in mature MK cultures. To investigate this, we sequenced 192 single cells from a D40 A1ATD1 (*35*) MK culture (99% CD41^+^ and 76% CD42^+^) using the Smart-Seq protocol. The increased sequencing depth allowed us to investigate possible cell surface–associated markers that could be used to purify the MKPs from mixed cultures.

**Fig. 2.**
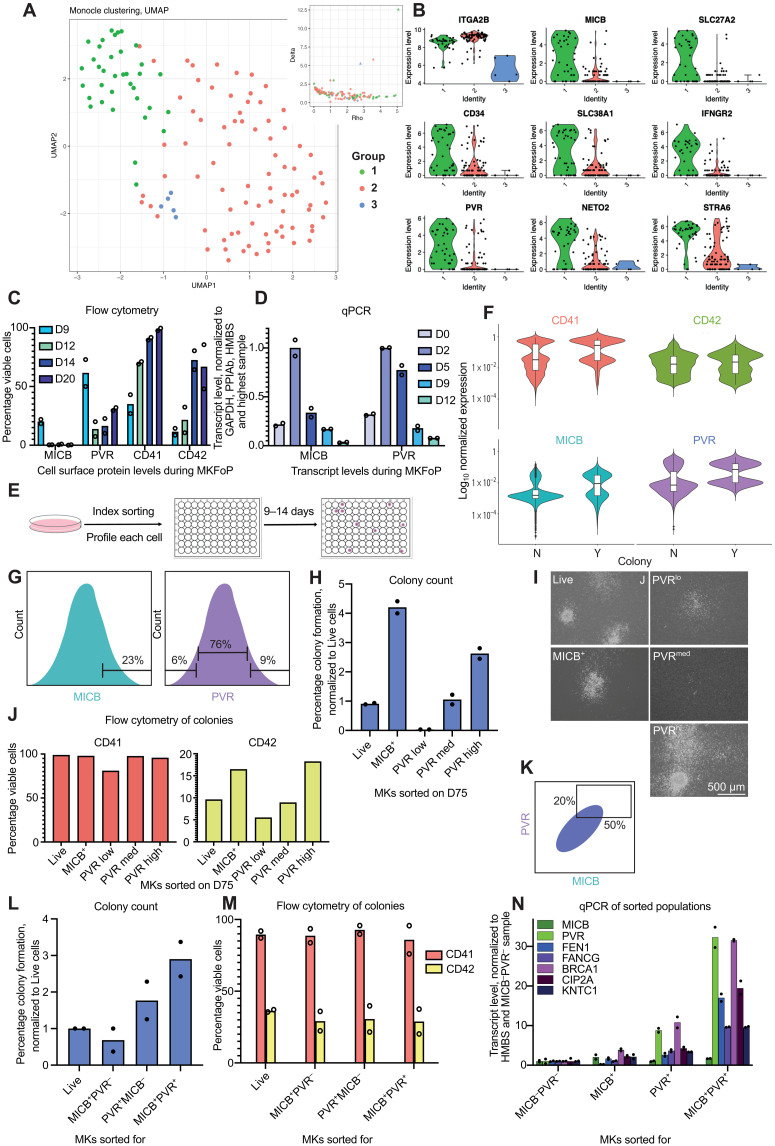
Identifying MKP surface markers by scRNA-seq. (**A**) UMAP of SMART-Seq scRNA-seq of a D44 A1ATD1 culture. Insert: Density clustering decision plot. (**B**) Normalized putative marker expression per cluster in (A). (**C**) Flow cytometry analysis of MICB, PVR, CD41, and CD42 expression during FFDK and QOLG3 MK differentiation. (**D**) qPCR analysis of *MICB* and *PVR* transcript levels during A1ATD1 MK differentiation. (**E**) Index sort schematic. (**F**) Index sort results: A1ATD1 MKs. Expression in cells that did not (N) and did form a colony (Y). CD41 (*n* = 7), CD42 (*n* = 7), MICB (*n* = 6), and PVR (*n* = 8). (**G**) Sorting scheme. (**H** to **J**) Colony counts (H), bright-field microscopy (I), and flow cytometry analysis (J) on D12 after D75 A1ATD1 MKs were sorted for MICB or PVR. (**K**) Sorting scheme. (**L** and **M**) Colony counts (L) and flow cytometry analysis (M) on D11 or D13 after D33 or D35 A1ATD1 MKs were sorted into CFU assays. (**N**) qPCR analysis of sorted D23 A1ATD1 MKs. Replicates are shown as points. For flow cytometry, viable cells were selected as DAPI negative and thresholds set on a corresponding isotype control for each sample. All colony counts were averaged per experiment (*n* = 2) and normalized to plated cell number and Live cell condition.

After data filtering and analysis (Materials and Methods), three clusters were identified using Monocle (*36*) (Fig. 2A). Transcript enrichment analysis allowed us to identify intermediate MKs that expressed *ITGA2B* at reduced levels (group 1), mature MKs with high *ITGA2B* expression (group 2), and apoptotic cells (group 3). We found the first cluster most interesting because of its reduced maturity. We performed gene set enrichment analysis on the top 25 markers of each cluster (Materials and Methods). All 44 enriched terms enriched in cluster 1 were involved in DNA replication, with E2F being the top enriched transcription factor (table S6). This indicated that this cluster is highly replicative and thus could contain MKPs.

We examined the transcripts enriched in the putative progenitor cluster and selected those that were highly expressed and cell surface associated as criteria for putative MKP markers (see Materials and Methods for details). Among the potential markers (Fig. 2B), we selected MHC class I–related peptide B (MICB) and poliovirus receptor (PVR). Details of their functions are included in Table 2. To investigate protein expression during differentiation, we carried out flow cytometry for cell surface MICB and PVR during MK differentiation (Fig. 2C). Both MICB and PVR were expressed by higher proportions of cells on D9 of differentiation and decreased as the MKs reached maturity on D20 (as demonstrated by CD41 and CD42 expression). To gain better resolution on expression levels before D9, transcript levels of the markers were assessed by quantitative polymerase chain reaction (qPCR) during early differentiation. *MICB* and *PVR* transcript levels peaked on D2 of differentiation and were reduced after this (Fig. 2D), again indicating that they could mark an early progenitor state.

To investigate whether these markers functionally define MKPs, we performed 20 index sorts (Fig. 2E and Materials and Methods). MICB and PVR both strongly enriched for cells that formed colonies (Fig. 2F). CD41 also strongly enriched for colony-forming cells, whereas CD42 had a much weaker effect. The results of these index sorts indicate that the cell surface expression of MICB and PVR is associated with MK colony formation ability, and thus, these two surface proteins could mark MKPs in bulk cultures.

To confirm this result, a colony-forming unit (CFU) assay was carried out (Fig. 1D). MICB^+^ (top 23%) or PVR^lo^ (bottom 6%), PVR^med^, or PVR^hi^ (top 9%) cells were sorted from a D75 A1ATD1 MK culture, which was >86% CD41^+^ (Fig. 2G). The sorted cells were plated in methylcellulose CFU assays and compared to live cells sorted without selecting for particular markers. Ten days later, colonies were scored (Fig. 2H). MICB^+^ cells formed more than four times more colonies than live-sorted cells, indicating that MICB marked MKPs. The level of PVR expressed also correlated with colony formation. PVR^hi^ cells formed more than twice the number of colonies in the control (Fig. 2, H and I). All colonies had consistent levels of CD41 expression when collected for flow cytometry analysis (Fig. 2J), indicating robust formation of MKs. Colonies derived from MICB^+^ or PVR^hi^ cells expressed higher CD42 than other colonies (Fig. 2J).

To ascertain whether sorting for both markers together would purify the progenitor population further, or whether both markers redundantly specified the same group of MKPs, cells were sorted for MICB and PVR in combination. The cutoffs were informed by the data acquired in the index sort (Fig. 2F) and the previous colony assay sorting for MICB and PVR individually. We selected the top 20% of PVR expressors and combined this with the top 50% of MICB expressors (Fig. 2K). MICB^+^PVR^−^ cells formed fewer colonies than live cells, implying that all MICB^+^ colony-forming potential is contained within the MICB^+^PVR^+^ fraction. Sorting for PVR enriched MKPs twofold, but adding MICB as a marker to PVR further enriched colony formation to threefold (Fig. 2L). Flow cytometry analysis of the colonies (Fig. 2M) demonstrated a similar mature MK production between conditions. We can conclude that adding MICB to PVR results in more efficient MKP purification from bulk MK cultures.

We then set out to measure the correlation of these sorted populations with putative MKPs as defined by the single-cell sequencing. A set of transcripts was selected from the progenitor cluster shown in Fig. 2A, and qPCR primers were designed. D23 A1ATD1 MKs were sorted for MICB and PVR expression, and progenitor-associated transcripts were quantified in these sorted populations by qPCR. Cells with high expression of either MICB or PVR alone expressed higher levels of progenitor-associated transcripts than control cells. Cells with high expression levels of both MICB and PVR showed even higher levels of progenitor-associated transcripts than either single-positive population (Fig. 2N). This first round of selection for MKP markers therefore allowed us to enrich MKPs by a factor of up to 3 (Fig. 2L) using the CD41^+^MICB^+^PVR^hi^ gating strategy.

### Further refining MKP markers: Single-cell snapshot of established culture

*MICB* and *PVR* were very poorly detected by 10x Genomics sequencing of the differentiation time course despite being well expressed on the protein level when measured by flow cytometry (fig. S4, D to F). We postulated that more highly expressed markers from the 10x dataset could be used to further refine the MKP panel. Working from the time-course UMAP and focusing on the region that we hypothesized to contain the MKPs (namely clusters 6, 15, and 34; see above and Materials and Methods), we selected the following markers: *ANXA1*, *HMMR* (CD168), *ITGAV* (CD51), and *FLT1* (VEGFR1) (fig. S4, G to J). A summary of the literature surrounding these candidates is provided in Table 2. The markers were all more highly expressed in clusters 6, 15, and 34 than the mature MKs (clusters 5, 11, and 48) on the time-course UMAP (Fig. 1G  and fig. S4). These markers and combinations thereof should allow us to pinpoint the location of the MKPs: Clusters 6 and 34 are marked by *HMMR* and *ITGAV* (including the small projection from cluster 6 that has the highest homology to in vivo human MKPs) and cluster 15 by *ITGAV* and *FLT1*. *ANXA1* was enriched in cluster 11, which is directly downstream on the velocity graph of cluster 15, toward the mature MKs (Fig. 1G and fig. S4). The cluster projecting from cluster 6 has the highest homology to in vivo human MKPs, and *HMMR* and *ITGAV* appear to be enriched in this area.

**Fig. 3.**
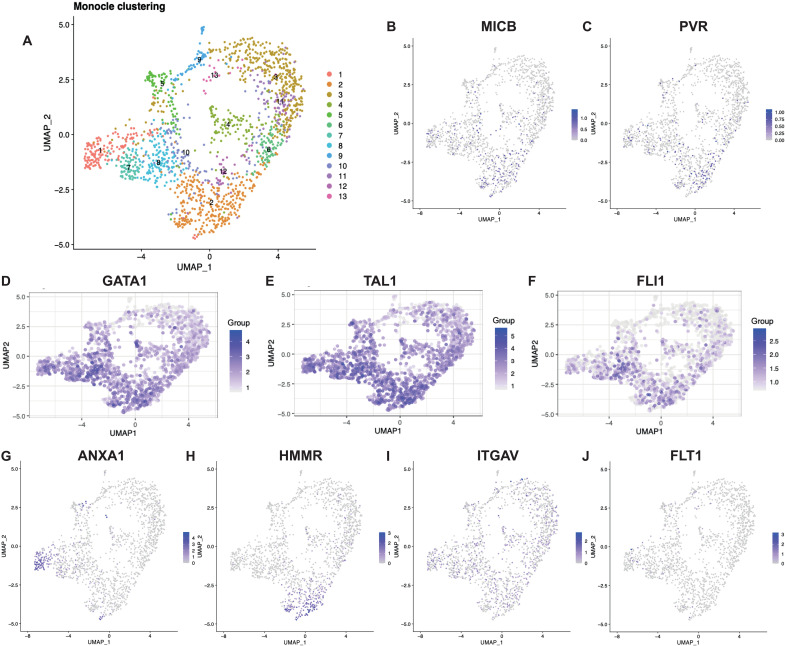
Single-cell sequencing of MKPs allows the selection of further candidate markers. (**A**) UMAP of 10x Genomics scRNA-seq of D27 A1ATD1 MKs sorted for the top 50% MICB, top 26% PVR, and bottom 33% CD42 expression. (**B** and **C**) UMAP of single-cell transform–normalized *MICB* and *PVR* expression. (**D** to **F**) UMAP of un-normalized *GATA1*, *TAL1*, and *FLI1* expression from lentiviral transgenes plotted on the gene expression UMAP. (**G** to **J**) UMAP of single-cell transform–normalized *ANXA1*, *HMMR*, *ITGAV*, and *FLT1* expression.

To determine whether these new markers were indeed expressed in MKPs, we purified CD41^+^ cells from a mature D27 A1ATD1 MK culture and sorted MICB^+^PVR^hi^ cells for sequencing using single-cell 10x Genomics workflow with transgene expression quantification. The 1661 cells that passed quality control were partitioned into 13 clusters and plotted on a UMAP (Fig. 3A). We identified clusters of interest using the same approach as described in Fig. 2A: enrichment of GO terms relevant to proliferative progenitors. The following clusters were identified as putatively containing MKPs: cluster 2 (enriched for cell division–associated GO terms and cell replication–associated transcripts such as *MCM3*, *MCM5*, and *MCM7*, *PCNA*, and *CENPW*), cluster 1 (increased transcript levels of *KIT*), and cluster 7 (enriched for cell division–associated GO terms). *MICB* and *PVR* were expressed by isolated cells from most clusters (Fig. 3, B and C), in keeping with the fact that the cells were selected for high expression of both these genes. Transgene sequencing showed higher expression of *GATA*, *TAL1*, and *FLI1* in all three clusters of interest (Fig. 3, D to F, and fig. S6F). We then looked at the four candidates (*ANXA1*, *HMMR*, *ITGAV*, and *FLT1*) identified from the UMAP time course (see Materials and Methods for candidate marker selection procedure): *ANXA1* marked cluster 1 and was also weakly expressed in clusters 7 and 8 (Fig. 3G). *HMMR* marked cluster 2 (Fig. 3H). *ITGAV* was broadly expressed at low levels (Fig. 3I), whereas very little *FLT1* expression was detected, apart from a few cells in cluster 1 (Fig. 3J). We concluded that one or combinations of all these surface proteins (ANXA1, CD168, CD51, and VEGFR1) together with MICB and PVR could refine our strategy to pinpoint MKPs within the culture system.

### Random forest feature selection identifies discriminative MKP markers

**Fig. 4.**
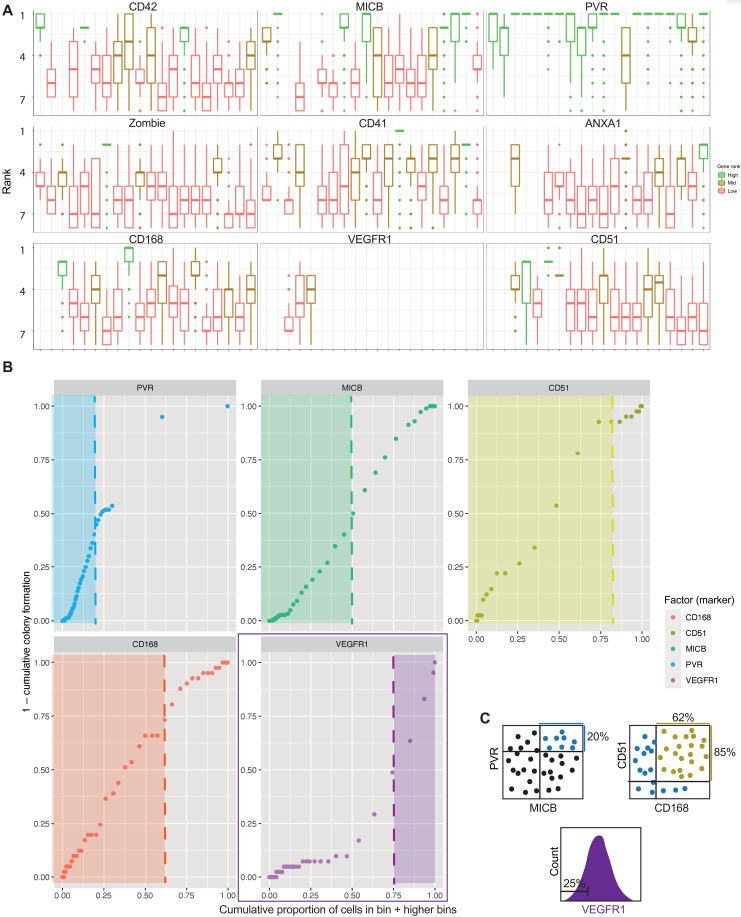
Identification of a panel of surface markers that can further purify MICB^+^PVR^+^ MKPs. (**A**) Twenty index sorts of A1ATD1 MKs. Rank indicates the first occurrence of a marker in a depth-first search of the decision trees making up the bagging model; genes with consistently low rank are discriminative for a given replicate. (**B**) Determining cutoffs for MKP markers based on all index sorts of A1ATD1 MKs. Outliers were excluded, and the 20 datasets were normalized and combined. The resulting dataset was then divided into 60 bins based on PVR expression and the colonies and noncolonies counted in each bin. This allowed the calculation of (1 − proportion colony formation per bin) (*y* axis). Cells were then ordered with decreasing PVR expression, and the proportion of cells with higher than this value of PVR is plotted on the *x* axis. The dataset was then subsetted on this expression level of PVR (top 20%), and the analysis was repeated for MICB, CD51, CD168, and VEGFR1. Areas with maximum colony formation are shown with shaded boxes. (**C**) Final sort strategy for MKPs. Cells are first purified as MICB^hi^PVR^hi^, then as CD51^hi^CD168^hi^, and lastly as VEGFR1^lo^.

To investigate which combination of ANXA1, CD51, CD168, and VEGFR1 could select for MKPs and therefore identify the area of the UMAPs in which the MKPs were found, 20 index sorts were carried out on A1ATD1 MKs. The data were augmented to obtain a 1:1 ratio of positive and negative observations (see Materials and Methods for details). Discriminative features were identified using the rank distributions from bagging analysis (Fig. 4A). The most distinguishing marker for colony formation was PVR (median rank 1 on 14 replicates) followed by MICB (median rank 1 on 4 replicates), confirming our previous colony formation results. CD41 and CD168 followed with median rank 1 on one replicate each. CD51 was median rank 2 on two replicates (Fig. 4A and fig. S5E). In contrast, ANXA1 performed poorly and did not appear to be associated with the MKP state. VEGFR1 was consistently ranked at the end of the selection table; however, it further purified populations selected by all other markers when used as a negative marker (*P* < 0.05 between colonies and noncolonies by Wilcoxon test on the entire, normalized, outlier excluded dataset).

To determine the cutoffs required for most selectivity, all datasets were combined after outlier exclusion and normalization. The combined dataset was binned into 60 bins and colonies and noncolonies scored in each bin, allowing the calculation of colony formation per bin. We then plotted the variable (1 − cumulative colony formation percentage) as a function of cumulative proportion of cells in each bin + bins with higher marker expression (Fig. 4B). At the bottom left of the graph are the cells with the highest expression level where 100% of cells form colonies. As we include cells with lower and lower levels of expression, the percentage of CFU decreases to the baseline corresponding to the bulk level, where all cells are included. The optimum cutoff of each marker is marked by the point of inflection of each graph. For positive markers, we use cells below the cutoff, but as VEGFR1 is a negative marker, we use the cells above the cutoff. As PVR was ranked as the first marker in the highest number of index sorts, PVR was plotted first (Fig. 4B). Selecting the top 20% of PVR-expressing cells selected most MKPs. The dataset was then subsetted to contain only cells expressing this level of PVR and further refined using the other markers, in increasing order of *P* value from a single index sort. Selecting the top 50% of MICB-expressing cells, the top 85% of CD51-expressing cells, the top 62% of CD168-expressing cells, and the bottom 25% of VEGFR1-expressing cells purified the highest proportion of total colonies. The final panel was subsequently tested on all datasets separately, outperforming all other marker panels generated by decision tree or random forest analysis (Fig. 4C).

**Fig. 5.**
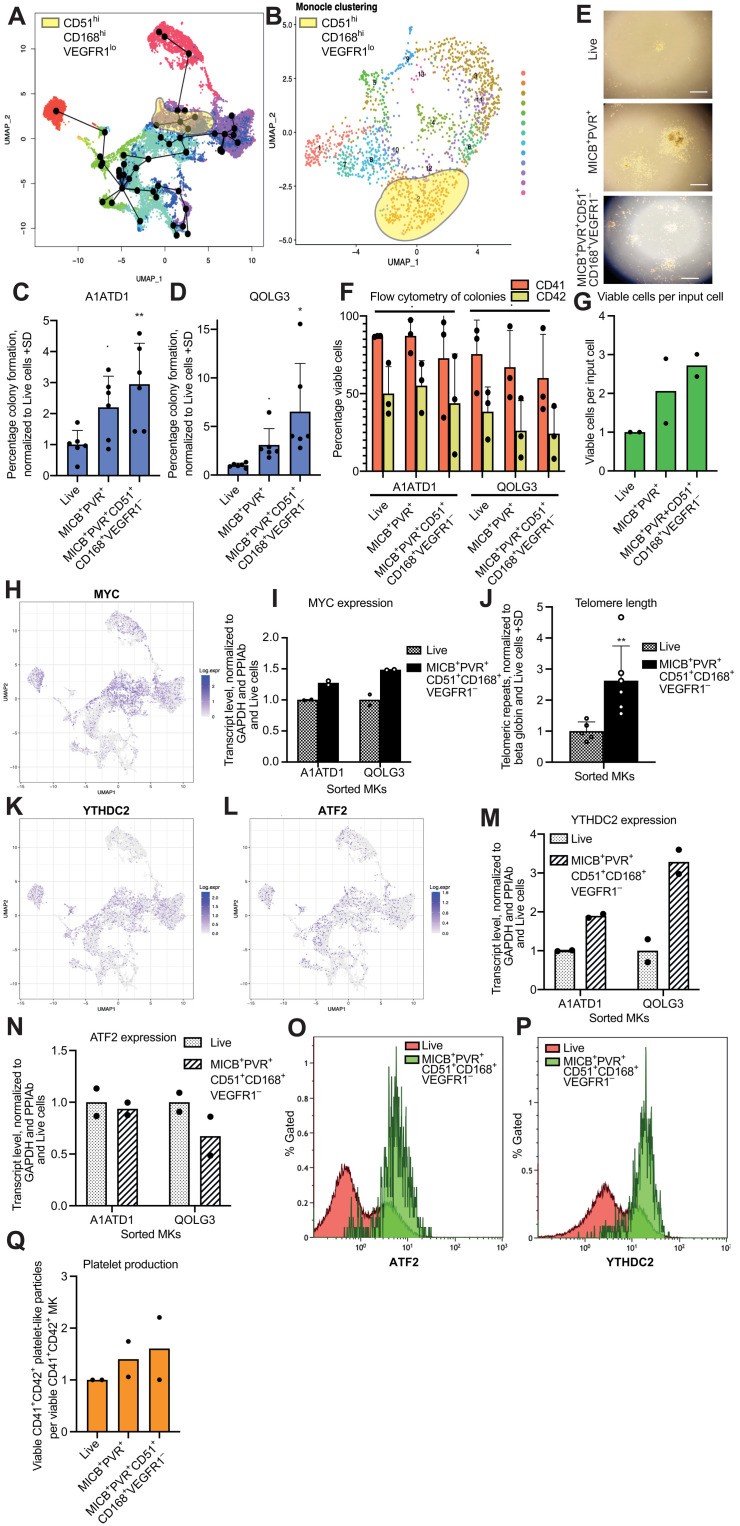
CD51, CD168, and VEGFR1 can further select MICB^+^PVR^+^ MKPs. (**A** and **B**) *ITGAV*^hi^*HMMR*^hi^*FLT1*^lo^ cells’ location on the trajectory plot (A) and MKP UMAP (B). (**C** and **D**) Colony counts from CFU assay of A1ATD1 (C) or QOLG3 (D) MKs sorted as MICB^hi^PVR^hi^ or MICB^hi^PVR^hi^CD51^hi^CD168^hi^VEGFR1^lo^. (**E** and **F**) Bright-field microscopy images (E) and flow cytometry analysis (F) of colonies. (**G**) Total viable cell count after one A1ATD1 and one QOLG3 CFU assay. (**H**) *MYC* expression levels on time-course UMAP. (**I**) qPCR analysis of *MYC* transcript levels. (**J**) qPCR analysis of telomeric repeats (D21 A1ATD1 and D34 QOLG3 MKs). Error bar, SD of three technical replicates, biological duplicates. (**K** and **L**) *YTHDC2* and *ATF2* expression on time-course UMAP. (**M** and **N**) qPCR analysis of *YTHDC2* (M) and *ATF2* (N) transcript levels. (**O** and **P**) Flow cytometry analysis of intracellular ATF2 (O) and YTHDC2 (P) levels in live and MICB^hi^PVR^hi^CD51^hi^CD168^hi^VEGFR1^lo^ D38 QOLG1 MKs. (**Q**) Flow cytometry analysis of calcein^+^ platelet-like particles 72 hours after MKs were sorted into basal medium. **P < 0.01, *0.05 < *P* < 0.01, and .*P* ≥ 0.05. Adjusted *P* value compared to Live cells by one-way analysis of variance (ANOVA) using Dunnett’s multiple comparisons test (C, D, and F) or two-tailed Mann-Whitney test (J). CFU counts were normalized to the total sorted cell number and the Live condition, shown as technical duplicates of biological triplicates. (I, M, N, and Q) D27 A1ATD1 and D34 QOLG3 MKs.

Using the gene expression data of *ITGAV*, *HMMR*, and *FLT1* (fig. S4C), we then pinpointed the MKPs on the time-course UMAP (Fig. 5A). These cells were located in clusters that lead to the mature MK cluster in trajectory analysis (Fig. 1J), expressed the highest *FLI1* levels among their time point (Fig. 1K), and included the region of cluster 6, which was most similar to in vivo MKPs (Fig. 1I).

A similar analysis was carried out on the MICB^+^PVR^+^ UMAP (Fig. 3), showing that the MKPs identified using this improved marker panel were found in cluster 2, which had the most proliferation-associated GO terms (Fig. 5B). Determining the surface marker expression of the MKPs allowed us to confirm their location on both UMAPs, pinpoint their emergence during differentiation, and gain an insight into which transcripts define the MKP state.

### Novel panel of surface markers reproducibly purifies MKPs from iPSC and primary cell cultures

We first investigated whether this new panel (MICB^hi^PVR^hi^CD168^hi^CD51^hi^VEGFR1^lo^) outperformed MICB and PVR alone for MKP selection using additional cell lines. MKs from two independent lines of iPSCs (A1ATD1 and QOLG3) were sorted. MICB^+^PVR^+^ cells showed increased colony formation in comparison to live-sorted cells (by a factor of 2 or more), and the addition of the new markers further increased colony formation in both lines by another factor of 2 to 3 (Fig. 5, C to E) (specificity and sensitivity of individual markers are shown in table S9). MK markers were similarly expressed under all conditions by flow cytometry of collected colonies (Fig. 5, F and G). Thus, combining CD51, CD168, and VEGFR1 with PVR and MICB can enrich MKPs by a factor of up to 6-fold in multiple iPSC lines. MKs derived from these progenitors have a surface marker profile that is identical to colonies from live-sorted MKs, demonstrating that these progenitors generate mature MKs.

To investigate whether the new marker panel can purify MKPs generated by other systems of MK differentiation, CD34^+^ cells were isolated from the leukocyte depletion filters from three healthy human adult blood donors. These were cultured in thrombopoietin and interleukin-1β (IL-1β) to differentiate them into mature MKs. On D5 of culture, each sample was sorted into two populations: live cells and live MICB^hi^PVR^hi^CD168^hi^CD51^hi^VEGFR1^lo^ cells. These populations were plated in colony formation assays in technical duplicate for each biological triplicate. On D13, colonies were counted, and MICB^hi^PVR^hi^CD168^hi^CD51^hi^VEGFR1^lo^ cells again formed more colonies than live cells sorted in parallel (fig. S5F). Thus, this new marker panel can be used to purify MKPs generated from adult human peripheral blood and from iPSCs.

### MKPs have specific biology compared to mature MKs

The transcription factor MYC has been reported to act as a key rheostat for the MKP state (*13*, *33*). In iPSC-directed differentiation protocols, MYC up-regulation leads to the efficient generation of MKPs, and its down-regulation is required for platelet production (*37*). MYC overexpression also generates immortalized MKPs, which are capable of producing mature platelet-producing MKs upon MYC down-regulation (*38*). We noted that *MYC* is up-regulated in the clusters leading toward the mature MKs on the time-course UMAP and down-regulated in mature MKs (Fig. 5H). We confirmed this by sorting the population of MKPs using the markers described above and quantifying *MYC* expression by qPCR in two independent iPSC lines (A1ATD1 and QOLG3). MKPs expressed higher levels of *MYC* than live-sorted cells (Fig. 5I), consistent with their more proliferative state.

Telomeres are protective repeat sequences found at the end of chromosomes. Telomeres shorten as cells divide, which is well documented in hematopoietic cells (*39*). Patients with shortened telomeres can suffer bone marrow failure (*40*). As MKPs maintain the forward programmed culture for up to 3 months, we hypothesized that they should have longer telomeres than mature MKs. To investigate this, we sorted A1ATD1 and QOLG3 MKs for live cells and MKPs and carried out qPCR on the resulting genomic DNA (gDNA) for telomeric repeats, comparing them to the β-globin locus, of which there should just be two copies per cell. In both cell lines, MKPs had longer telomeric repeats than live-sorted cells from the same culture (Fig. 5J), indicating that these MKPs may have lengthened telomeres, supporting their long-term proliferative potential.

As MKPs exhibit a different transcriptional signature to mature MKs, we hypothesized that they could also express different levels of epigenetic modifiers. We found that *YTHDC2* and *ATF2* were both more highly expressed in the MKP cluster (Fig. 5, K and L). YTHDC2 is an RNA helicase that promotes the translation of m^6^A-modified mRNA (*41*) by attaching it to the ribosome (*42*) and is essential for meiosis (*43*). ATF2 is a histone acetyltransferase specific for H2B and H4 (*44*) and regulates γ-globin expression by histone deacetylase recruitment (*45*). We analyzed the expression of *YTHDC2* and *ATF2* in the live cells and sorted MKPs from A1ATD1 and QOLG3 MK cultures and confirmed that *YTHDC2* transcript levels were increased in MKPs, whereas *ATF2* transcript levels were unchanged (Fig. 5, M and N). However, when we analyzed the expression of both these proteins by intracellular flow cytometry, both were increased in MKPs versus bulk MKs (Fig. 5, O and P). Therefore, MKPs express higher levels of both these epigenetic modifiers than bulk MKs. Demonstrating their functionality, MKPs purified using the new surface marker panel also produced similar levels of viable platelet-like particles to MICB^+^PVR^+^ MKPs or live-sorted cells (Fig. 5Q).

### Optimization of culture condition for MKP production in vitro

Identifying specific markers for MKPs provided us with the tools to optimize culture conditions to promote MKP creation in early culture stages and the maintenance, proliferation, and maturation of MKPs during long-term culture. We first addressed the early stage of culture, carrying out MK differentiation under two oxygen (O_2_) concentrations: ambient (21%) and low (5%) O_2_. Low O_2_ has been shown to be beneficial for the culture of bone marrow–derived MKPs (*46*). During MK differentiation from HSCs, low O_2_ was beneficial at early stages, whereas ambient O_2_ was optimal for later stages (*47*). We used three different concentrations of each of the cytokines stem cell factor (SCF) and TPO (thrombopoietin). The percentage of cells expressing CD41, CD42, and progenitor-associated markers was monitored along the differentiation time course in this matrix of conditions.

**Fig. 6.**
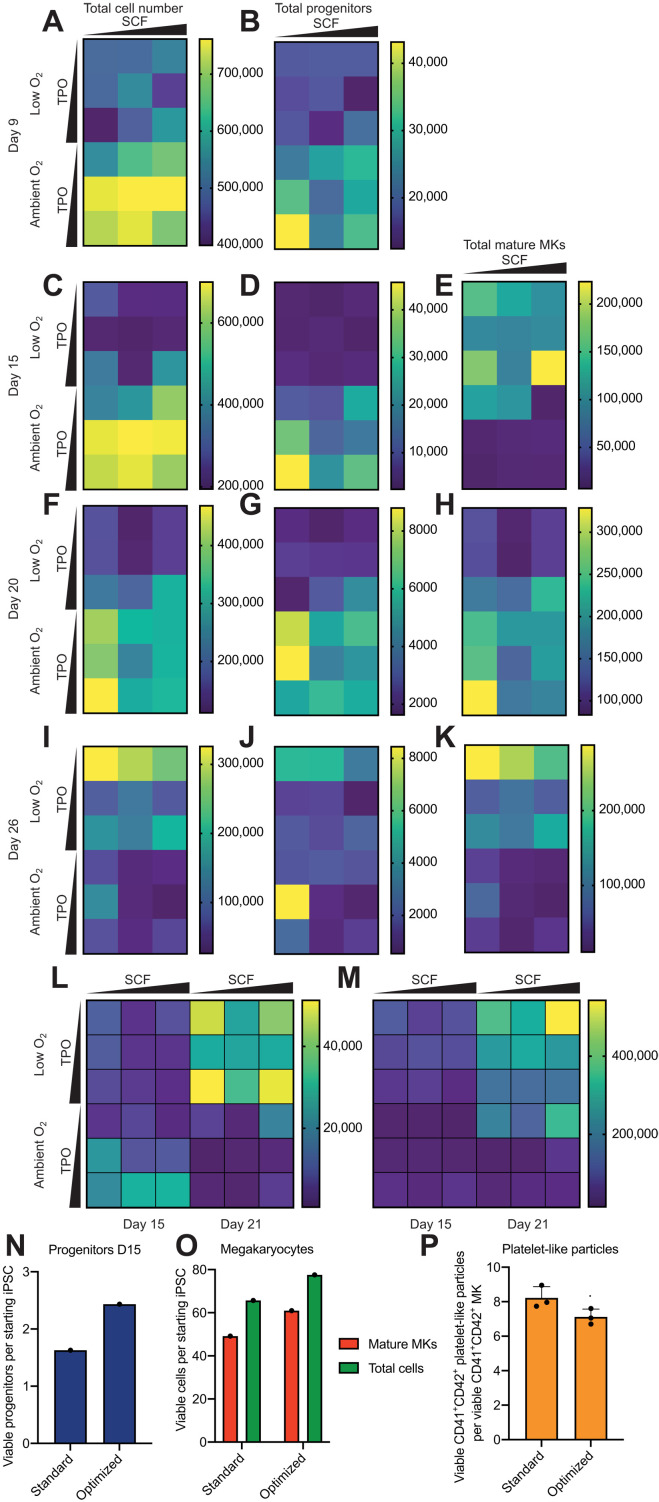
Optimizing culture conditions for MKP production in vitro. (**A**) MK differentiation of QOLG3 iPSCs was carried out in varying concentrations of SCF (12.5, 25, and 50 μg/ml), TPO (10, 20, and 40 μg/ml), and O_2_ [5% (low) and 21% (ambient)]. Total viable cell counts per well (as defined by FSC/SSC and DAPI negativity, quantified by using flow count beads and flow cytometry). (**B**) Total MICB^hi^PVR^hi^CD51^hi^CD168^hi^VEGFR1^lo^ cells (“progenitors”) were quantified under each condition. (**C**) As (A), D15. (**D**) As (B), D15. (**E**) Total CD41^+^CD42^+^ cells (“mature MKs”) per well. (**F** to **K**) Cell number, progenitor number, and mature MK number on D20-D26. (**L**) Total progenitor count in A1ATD1 MKs on D15 and D21. (**M**) Total mature MKs per well in A1ATD1 on D15 and D21. (**N** and **O**) QOLG3 iPSCs were differentiated under standard and optimized cytokine and O_2_ concentrations. Number of progenitors (N), mature MKs, and total cells (O) generated per starting iPSC under each condition. All samples gated on 21% O_2_, SCF (25 μg/ml), and TPO (20 μg/ml). (**P**) Flow cytometry analysis of platelet-like particles 24 hours after D52 QOLG3 MKs produced under standard and optimal conditions were plated in basal medium. Samples were stained with calcein AM for platelet-like particle viability, DAPI for cell viability, and CD41 and CD42. Calcein^+^CD41^+^CD42^+^ platelet-like particles and DAPI^−^CD41^+^CD42^+^ MKs were quantified. +SD shown for three technical triplicates. .*P* ≥ 0.05 when compared with standard conditions by two-tailed Mann-Whitney test.

More viable cells (Fig. 6A) and more progenitors (Fig. 6B) were produced on D9 in ambient O_2_ concentrations, and these conditions retained the highest viable cell number (Fig. 6C) and the most progenitors (Fig. 6D) on D15. Low O_2_ conditions caused the most MK maturation (Fig. 6E).

On D20, the condition that had yielded the most MKPs at D9 and D15 (ambient O_2_, high TPO, and low SCF) now yielded the most MKs (Fig. 6H), showing that early generation of MKPs resulted in the later generation of mature MKs. Ambient O_2_ still had the most MKPs at D20 (Fig. 6G); however, low O_2_ promoted more MKP generation after D20 (fig. S6G). This increased the production of MKPs in low O_2_ and low TPO after D15 translated into the highest number of mature MKs by D26 (Fig. 6K), again demonstrating that increasing MKP generation early in the culture increased mature MK yield later on. The experiment was repeated with A1ATD1 MKs, and again, more MKPs were produced on D15 at ambient O_2_, low SCF, and high TPO, but later on in culture, more MKPs were produced in low O_2_ (Fig. 6, L and M).

We then carried out an “optimized culture” where cells were kept at ambient O_2_, low SCF, and high TPO up until D15 and thereafter in low O_2_ and low TPO. By D15, the optimized culture conditions improved MKP production to 1.5-fold (Fig. 6N) and mature MK production to 1.23-fold production under standard conditions (Fig. 6O). MKs produced using optimized conditions produced similar platelet-like particle numbers per MK as MKs produced under standard conditions (Fig. 6P). In conclusion, the panel of surface markers identified as part of this study allowed us to quantify MKPs in culture and thus optimize cytokine and oxygen concentrations to maximize MKP production, thereby postponing culture exhaustion, maximizing MK output, and driving down the cost of platelet production.

## DISCUSSION

In this study, we have mapped MK differentiation at high resolution from iPSCs to mature MKs, generating an overview of the cell fates acquired during differentiation and demonstrating that this protocol accurately generates MKPs and erythroid progenitors, which mirror their in vivo counterparts in human bone marrow, peripheral blood, and spleen. Microarrays have been used to analyze immortalized MK lines (*48*), and RNA-seq has been used to analyze initial MK differentiation from ESCs (embryonic stem cells) (*18*) to compare MKs differentiated in vitro to MKs from cord blood–derived CD34^+^ HSCs (*17*). However, this is the first time that in vitro–produced MKs have been directly compared to in vivo progenitors. This study demonstrates that, although in vitro MK differentiation appears to mirror in vivo development from pluripotent cells to mesoderm, hemogenic endothelium, and, lastly, commitment to the megakaryocytic lineage, the cells do not pass through a state resembling HSCs or MPPs in vivo. Directed differentiation protocols often shepherd cells through a cell state characterized by the expression of CD31, associated with hemogenic endothelium (*13*, *14*), and CD34, which marks HSCs (*13*, *49*). This study indicates that transcription factor–mediated forward programming may accelerate differentiation, proceeding directly to more committed progenitors.

Single-cell sequencing has enabled us to pinpoint the transcriptional epi-states through which cells must transit to become mature MKs, using single-cell trajectory analysis to identify clusters that correspond to MKPs and persist into late cultures. Confirming the results of previous studies (*22*) and consistent with the crucial role of FLI1 during megakaryopoiesis in vivo (*23*) and MKP differentiation from HSPCs in vitro (*50*), we observe increased *FLI1* expression in the clusters leading to mature MKs by trajectory analysis. Combining index sorting for key marker candidates and machine learning on the resulting high-dimensional datasets, we have identified a panel of novel surface markers that can be used to purify MKP cells as they differentiate from human iPSCs. Previous studies described panels to purify MKPs from MEP cultures (*9*). This study describes the first surface marker panel allowing human MKP purification from iPSC- and peripheral blood HSC–derived MK cultures, their quantification, and the optimization of their production.

The set of markers described here also allowed insights into key aspects of MKP biology. We observed an increase in *MYC* expression in MKPs, supporting the results of other groups that describe the down-regulation of MYC during MK maturation (*13*, *37*, *38*). Increased levels of MYC also explain the increased replicative capacity of MKPs. In addition, we also observed an increase in telomere length in purified MKPs, which may underlie their increased proliferative potential. As hematopoietic cells divide, either during aging or cytokine-supplemented culture, their telomeres shorten (*39*). This shortening can result in bone marrow failure, such as that seen during dyskeratosis congenita, aplastic anemia, and Fanconi anemia (*40*). MKPs may require lengthened telomeres to support their replication for the extended periods of in vitro culture observed here.

Purification of MKPs using the marker panel described in this study also allowed us an insight into the epigenetic modifiers expressed by MKPs. This is an indirect assessment of potential differences between MKPs and mature MKs, which would need to be assessed formally in further studies. We have described an increase in ATF2, a histone acetyltransferase for H2B and H4 (*44*), in MKPs in comparison to cells in the bulk culture. Histone deacetylase inhibitors have been previously described to increase human CD34^+^ HSPC differentiation to MKPs (*50*), indicating increased histone acetylation in the MKP state. ATF2 has been shown to up-regulate the expression of γ-globin in erythroid progenitors (*45*), implying its involvement in erythroid differentiation. As MKPs are derived from the erythroid cluster of our time-course dataset, this could further underline the association of ATF2 with the MKP state. YTHDC2 is an RNA helicase that binds m^6^A-modified RNA (*41*) and attaches them to the ribosome, “fast-tracking” their translation (*42*). Fewer granulocyte/erythroid/macrophage/MK progenitors were formed by m^6^A-depleted HSPCs (*51*), indicating that m^6^A may be important for their differentiation. *MYC* RNA is marked by m^6^A in leukemic cells, and m^6^A reduction reduces MYC (*51*) by reducing the half-life of its RNA (*52*). Therefore m^6^A is crucial for MYC expression. The increased levels of m^6^A binder YTHDC2 observed here may be linked to the increased *MYC* levels we observe in MKPs; YTHDC2 may stabilize *MYC* transcripts and expedite their translation.

Optimizing cell culture conditions to increase cell output and diminish the cost of goods remains one of the major challenges in translating iPSC-derived cellular products to the clinic (*21*, *53*). Using the set of MKP markers identified here, we found that adjusting oxygen and cytokine conditions can increase MKP generation, maximizing mature MK generation. Ambient oxygen has been demonstrated to promote the later stages of MK differentiation from HSCs (*47*). Only later hematopoietic intermediates are generated in our differentiation system, explaining why ambient oxygen promotes optimal initial differentiation in our system. More progenitors were produced in later cultures under low oxygen tension in this study. Low oxygen tension has been shown to allow the culture of MKPs from human bone marrow (*46*), implying that low oxygen can retain the MKPs already present in the cultures at this time. The optimized conditions identified here could thus allow the early expansion of MKPs and their retention over time.

In conclusion, the bioinformatic and biological tools illustrated in this study are broadly applicable to other stem cell differentiation systems. They allow the identification of specific surface markers for cellular products, promoting a better biological understanding of stem cell types generated in vitro, allowing their comparison to in vivo counterparts and the optimization of their production for the progress of novel stem cell–derived therapeutics toward the clinic.

## MATERIALS AND METHODS

### MK forward programming

iPSCs [A1ATD1 (*35*), HPSI1113i-bima, HPSI1113i-qolg_1, HPSI1113i-qolg_3, and HPSI0813i-ffdk_1] were seeded at single-cell density on vitronectin (VTN-N; Thermo Fisher Scientific, A14700)–coated plates (*17*). One day later, they were lentivirally transduced with *GATA1*, *TAL1*, and *FLI1* in the presence of protamine sulfate (10 ng/ml; Sigma-Aldrich, P4505) in Dulbecco’s modified Eagle’s medium/F12 (Gibco, 11330-03), 0.05% NaHCO_3_ (Gibco, 25080094), l-ascorbic acid (65 mg/liter; Sigma-Aldrich, A8960), 1× ITS (insulin, transferrin, selenium) (Gibco, 414000450), bone morphogenetic protein 4 (10 ng/ml; Bio-Techne, 314-BP), and fibroblast growth factor 2 (20 ng/ml; Bio-Techne, 233-FB). The following day, cells were washed in phosphate-buffered saline (PBS), and the medium was replaced without virus. On D3, the cells were washed in PBS and transferred to CellGenix GMP SCGM (Good Manufacturing Practice grade Stem Cell Growth Medium) (CellGenix, 20802-0500) supplemented with SCF (25 ng/ml; Gibco, PHC2116) and TPO (20 ng/ml; Bio-Techne, catalog no. 288-TP). On D10, the cells were removed from the plate using TrypLE Express (Gibco, 12604021), centrifuged in PBS, and replated in uncoated tissue culture plates in CellGenix GMP SCGM supplemented with SCF (25 ng/ml) and TPO (20 ng/ml). The culture was maintained at a density of 0.5 × 10^6^ to 2 × 10^6^ cells/ml, and cells were fed with SCF and TPO every 2 to 3 days by half medium exchange.

### MK differentiation from peripheral blood CD34^+^ cells

CD34^+^ hematopoietic progenitors were obtained from leukapheresis material provided by the NHS Blood and Transport (NHS BT, Cambridge). Peripheral blood mononuclear cells (pbMNCs) were isolated from peripheral blood using Ficoll-Paque gradient separation. CD34^+^ hematopoietic progenitors were extracted from pbMNCs using a CD34 MicroBead Kit (Miltenyi Biotec, 130-046-702) for magnetic separation following the manufacturer’s protocol. Purity of samples was assessed using flow cytometry with a CD34-phycoerythrin antibody (Beckman Coulter, A07776). All samples demonstrated greater than 95% CD34 expression.

Peripheral blood MKs (pbMKs) were derived from CD34^+^ progenitors as follows. Centrifuged CD34^+^ cells were resuspended in pbMK medium: SCGM, TPO (50 ng/ml), and IL-1β (5 ng/ml; CellGenix, 1411-050). Cells were plated into a 12-well plate at a density of 1 × 10^5^ cells/ml of pbMK medium and incubated at 37°C/5% CO_2_. On D3, 1 ml of fresh 1× pbMK medium was added to each well of pbMKs. On D5, single pbMKs were sorted using a FACSAria Fusion (BD Biosciences) into SCGM, and the cell concentration was adjusted to 1 × 10^4^ cells/ml. pbMKs were then added to 2.4 ml of nonenriched MethoCult (STEMCELL Technologies, H4535) supplemented with TPO (250 ng/ml), IL-1β (20 ng/ml), and 1× penicillin/streptomycin (PenStrep; Gibco, 15140122) in 300 μl of SCGM, and 1.4 ml of MethoCult-pbMK suspension was deposited in duplicate wells of a SmartDish (STEMCELL Technologies, 27370) and incubated at 37°C/5% CO_2_ for 8 days. Colonies were counted using a Primovert optical microscope (Zeiss). Following colony counts, pbMKs were liberated from the MethoCult by dilution in 10 ml of RPMI 1640 media (Gibco, 21875034) and centrifugation at 120*g* for 8 min. pbMKs were resuspended in 300 μl of basal media and stained with CD42a-APC (allophycocyanin) and CD41a-APC-H7 antibodies for 20 min before analysis using a Gallios flow cytometer (Beckman Coulter, A94303).

### 10x Genomics single-cell sample preparation

Cells were dissociated at 24-hour intervals during the first 10 days of forward programming and washed twice in ice-cold PBS. D15 or D20 cells were counted and collected into ice-cold PBS before being washed once more in ice-cold PBS. Cells were fixed in methanol, following the 10x Genomics demonstrated protocol for scRNA-seq (CG000136). They were rehydrated and submitted to the Cancer Research UK Cambridge Institute Genomics Core Facility for library preparation using 10x Genomics Chromium Single-Cell V(D)J Reagent Kit (protocol: CG000086 Rev H, chemistry 5’ v1.0: PN-1000014, PN-120236, and PN-1000020) with custom enrichment substituting the V(D)J primers for primers amplifying *GATA1*, *TAL1*, and *FLI1* transgenes (table S1 and fig. S1C). Progenitor cells were stained and sorted as previously documented before being submitted for library preparation. A gene expression library and two enriched libraries (one for *GATA1*+*FLI1* and one for *TAL1*) were generated for each sample.

This experiment was sequenced in three batches; the library preparation was performed in two batches. The first run included samples D0_1, D1_1, D2_1, D3_1, D4_1, D5_1, D6_1, D7_1, D8_1, D9_1, D10_1, D15_1, and D20_1. The second run included all samples from the first run, except D8_1 and D9_1, which were replaced by D8_2 and D9_2. In addition, D5_2 was included as a bridging sample to allow the evaluation of batch effects. Two new samples, D0_single2 (D0_s2: iPSCs plated as single cells with ROCK inhibitor) and D0_time-course2 (D0_t2: iPSCs plated with ROCKi), were also included. All sequencing data were generated using a NovaSeq6000. The first and second sequencing runs were performed with the following parameters: Read1: 26 base pairs (bp), Index1: 8 bp, and Read2: 91 bp. The third run included deeper sequencing of D3_1, D5_2, D8_2, D10_1, and D20_1 as well as all enriched libraries and was performed using PE150.

### Time-course 10x Genomics scRNA-seq analysis

Quality checks, alignment, and protein-coding gene quantification were performed using Cell Ranger v3.1.0; samples from the same biological batch but different sequencing runs were quantified separately. To maintain consistency across sequencing runs in the time-course dataset, reads from the third sequencing run were aligned single-end with the R2 strands clipped to 91 bp; in total, 72,310 cells were detected by Cell Ranger.

Next, further quality control was performed on the cells (fig. S2). The distribution of number of features per cell, the sequencing depth per cell, and the number of cells per sample varied between samples. Consequently, the resolution of the time course varied over time. The logarithmic relationship observed between sequencing depth and number of detected features per cell suggested that sequencing saturation was not reached.

Features (genes) expressed in fewer than three cells were excluded from downstream analysis; 23,949 genes were retained. Cells with <1000 detected genes, >60,000 unique molecular identifiers (UMIs), or >10% reads incident to mitochondrial genes were discarded, leaving 22,236 cells for downstream analysis (table S2). The dataset was normalized using sctransform.

UMAP of the filtered and normalized data (fig. S2) was used to assess the extent of batch effects between cells from different library batches and different sequencing runs. No clear separation was observed between cells from different sequencing runs, and while separation was observed between the library batches, the observed differences were likely the consequence of the overrepresentation of certain time points in either library batch. On the basis of these results, no batch correction was deemed necessary.

UMAPs colored by raw and normalized sequencing depth were inspected to check whether the heterogeneity observed in the dimensionality reduction was confounded with sequencing effects. Raw sequencing depth was uneven across the UMAP, but normalization reduced this variation across the cells. Normalized mitochondrial and ribosomal protein-coding expression levels were plotted on the UMAP, and regions generally expressed relatively high levels of either but not both.

After normalization using sctransform, clustering was performed with Seurat v3.1.4 [SLM algorithm (*85*) on the five nearest neighbor graph], Monocle v2.14 [density peak–based clustering on a t-SNE (t-distributed stochastic neighbor embedding) dimensionality reduction], and PCA followed by k-means, on the top *x* = (500, 1000, 2000) most abundant genes. Element-centric similarity (*76*) was used to assess the stability of the clustering with regard to the number of genes. The Monocle clustering on the 1000 most abundant genes, which comprised between 60 and 90% of counts in cells across samples, displayed the highest stability; these genes were subsequently used for dimensionality reduction using PCA and UMAP (created on 30 PCs, selected after inspection of elbow plot). The stability of the clustering, in terms of number of clusters, was assessed using the proportion of ambiguously clustered pairs (*77*). Cells from each sample are often clustered together in a small number of clusters, while individual clusters often extend beyond a single time point sample (fig. S2M). Marker genes for each cluster were identified using the ROC test in Seurat; genes with |ln(FC)| > 1 were considered for the ROC test; the selected markers for each cluster were the top 25 genes ranked by discriminative power (table S3).

Gene set enrichment terms were calculated with g:profiler (*78*), using the 25 genes with the highest power in the ROC test, with all genes returned by sctransform across all clusters used as background (table S4). A blacklist of biologically unrelated annotation terms was applied to avoid inflated similarities. Enrichment terms with g:SCS-adjusted *P* values of <0.05 were further explored. The cell cycle phase of cells was computationally inferred using the CellCycleScoring function in Seurat, using an annotated list of cell cycle–associated genes (*79*).

Pseudotime trajectories were calculated using Monocle on a DDRTree dimensionality reduction on the Pearson residuals of the abundant genes with parameter max_components = 2 and separately using Slingshot v1.4 (*28*) with default parameters on the original UMAP representation. Temporally varying genes were identified with quadratic regression of each gene’s expression on the Slingshot pseudotime variable. The heatmap was created using the 100 most significant genes, where, for ranking purposes, the smallest of the two *P* values for the linear term and the quadratic term was used for each gene.

### In vivo 10x Genomics scRNA-seq integration

An in vivo 10x Genomics scRNA-seq dataset of human HSPCs from the bone marrow, peripheral blood, and spleen of two donors was used to assess similarities between in vivo hematopoietic intermediates and in vitro MK differentiation using our optimized protocol. We used as starting point the count matrix with raw expression levels and metadata with annotated cell type identities (*27*). On the basis of the inspection of distributions of sequencing depth, number of detected features, and proportion of UMIs assigned to mitochondria and ribosomal genes (MT% and RP%, respectively), cells with >1000 features, <10% MT UMIs, and >20% RP UMIs were retained for further analysis, totaling 13,099 cells for donor 1 and 17,350 cells for donor 2. After normalization with sctransform, we used the dimensionality reductions, PCA, and UMAP (created on the 30 first PCs based on elbow plot of variance per component) to assess the consistency between patients. Substantial batch effects were observed between the two donors; consequently, normalization and analysis of the data were performed separately per donor to preserve donor-specific characteristics. Subsequently, only cells annotated as MEP, HSC/MPP, MKP, or erythroid progenitor were used for downstream analysis, leaving 9205 donor 1 cells and 12,214 donor 2 cells.

To identify marker genes for the annotated cell types, the ROC test in Seurat was used with a minimum fold change of 2.0; taking the union of positive markers across each cell type in both donors, 101 discriminative genes were detected. Random forest models (*80*) were trained separately per donor using the expression of these 101 genes and the annotated cell types as output (using the randomForest R package, version 4.6-14). Decision trees (100) were used in the random forest models; the convergence of the models was assessed using training error versus the number of trees; the models converged after 40 trees. The stability of the random forest models was assessed using 10-fold cross-validation; for the random forest trained on donor 1, the distribution of accuracies was high (minimum of 92.7%, median of 93.4%, maximum of 95%, and SD of 0.7%), and the random forest trained on donor 2 was similarly accurate (minimum of 90.3%, median of 91.3%, maximum of 93.4%, and SD of 0.9%).

Next, using the random forest model, we predicted the cell types for the 10x Genomics scRNA-seq time course. A cell is assigned to the cell type with the largest probability; by applying a minimum probability threshold, uncertain cell type predictions can be avoided. To determine the probability threshold, the percentage of D0 cells assigned to any cell type was used as criterion; the lowest probability threshold that ensured <1% of D0 cells was assigned to any type (as D0 cells should be in the pluripotent state and not represented in adult human bone marrow, spleen, or peripheral blood). For donor 1, the threshold was 0.53, and for donor 2, the threshold was 0.67.

### Sample preparation and Smart-Seq of mature MK culture

Single cells from a D44 A1ATD1 MK culture were sorted into two 96-well plates containing 2.3 μl of 0.2% Triton X-100 with 1× ribonuclease inhibitor and frozen at −80°C before library preparation. Reverse transcription was initiated with oligo-dT primers and SuperScript II reverse transcriptase (Thermo Fisher Scientific, 18064022) and supplemented with ERCC RNA Spike-In. The cDNA was then PCR-amplified for 21 cycles using the KAPA HiFi Hotstart ReadyMix and IS primers (Kappa Biosystems, KK2601). After purification of the amplified cDNA, the indexed library was made using the Illumina Nextera XT DNA kit. The libraries from 96 cells were pooled, and quality was checked on a Bioanalyzer high-sensitivity DNA chip (Agilent, 5067-4626) before sequencing on one lane of a HiSeq 4000 (Illumina).

Quality control of raw reads was performed using FastQC v0.11.3 and summarized with MultiQC v1.8. Checks included the variability of sequencing depth and distribution of GC content. Samples were aligned to the GRCh38.p13 reference genome with STAR v2.7.3a (default parameters) (*81*) using Ensembl v98 annotations. Protein-coding gene quantification was performed using featureCounts v2.0 (default parameters). Scatterplots of sequencing depth versus the number of detected features per cell were visualized; cells with fewer than 200,000 reads were excluded, leaving 123 cells for downstream analysis. Normalization of raw gene abundances was performed using sctransform (*82*).

Across the 192 samples, the distribution of raw sequencing depth was wide (fig. S5); after normalization and cell filtering, the distribution narrowed. We infer that sequencing saturation was reached for this experiment based on the lack of linear or logarithmic relationship between sequencing depth and number of features. We conclude that the observed heterogeneity in the data is not primarily dependent on the variation in sequencing depth.

To select the number of abundant genes, cluster stability with regard to gene expression was assessed using element-centric clustering. Using the density decision graph (Fig. 2A, inset), we identify only three cells that exhibit high density and are distant from cells of higher density; these cells are subsequently assigned as cluster centers (*83*).

Subsequently, the 2000 most abundant genes, accounting for between 60 and 80% of counts, in a majority of cells, were used for PCA and UMAP (created on the first 30 PCs that were used after inspection of elbow plot), as well as clustering. Two clustering approaches were compared side by side: graph modularity optimization–based clustering with Seurat v3.1.4 (*84*) and density peak–based clustering with Monocle 2.14 (*36*). A nearest neighbor graph with shared nearest neighbor weighting of the edges was created with Seurat using the first 30 PCs, with *k* = 20 neighbors; this was used for clustering using the SLM algorithm (*85*). Clustering was separately performed with Monocle v2.14, using a t-SNE dimensionality reduction and the density peak algorithm (*83*). Monocle was configured to find the same number of clusters (3) as Seurat (for the latter, detected with default resolution parameter).

To identify cell surface markers that could be used to purify MKPs by flow cytometry, marker genes for each cluster were identified using the ROC test in Seurat. Only genes with |ln(FC)| > 1 were considered for the ROC test, and the final markers for each cluster were the top 20 positive markers per cluster, ranked by discriminative power, which were used for gene set enrichment analysis. The list of up-regulated genes in the putative MKP population was filtered for protein products that locate to the plasma membrane (those that had plasma membrane–associated GO terms). In this way, a list of 23 genes was generated: *CDCA5*, *PRC1*, *FEN1*, *ERCC6L*, *KNTC1*, *BRCA1*, *ORC1*, *CIP2A*, *FANCG*, *HMMR*, *POLE*, *GAPT*, *MICB*, *FANCI*, *ORC6*, *IFNGR2*, *SLC27A2*, *CD34*, *SLC38A1*, *NETO2*, *STRA6*, *KDR*, and *PVR*. Those without reliable flow cytometry antibodies were excluded. Antibodies for BRCA1, IFNGR2, KDR, CD34, CD44, CIP2A, PVR, and MICB were tested by fluorescence-activated cell sorting, but only MICB and PVR were taken forward. qPCR primers were designed for the other targets.

### Sorted MKP 10x Genomics scRNA-seq

A D27 A1ATD1 MK culture was sorted for cells expressing PVR (top 26%) and MICB (top 33%). Live cells were subjected to 10x Genomics library preparation, generating separate libraries for gene expression and transgene expression as detailed previously. Prealignment quality control was performed analogously to the smart-seq2 analysis; subsequently, the 10x Genomics GRCh38 v3 reference genome was used for alignment and protein-coding gene quantification with Cell Ranger v3.1.0; 1899 cells were detected. The number of features and counts varied across cells; MT and RP distributions before filtering displayed long tails (fig. S6, A to E). The logarithmic relationship between the number of UMIs and the number of features detected per cell suggested that sequencing saturation was not achieved. After quality control and filtering, the distribution of counts per cell was narrower. UMAPs of MT and RP indicated that groups of cells have a high proportion of either MT or RP but not both.

Features expressed in fewer than three cells were excluded from further analysis; 16,660 genes were retained. Distributions of sequencing depth, number of detected features, and percentages of MT and RP incident reads per cell were inspected; on the basis of these distributions, cells with <1000 unique features or >10% MT reads were discarded; 1661 cells were retained for downstream analysis.

Sctransform was used for normalization of expression levels across cells; on the basis of the element-centric clustering similarity across clustering methods, the 2000 most abundant genes, accounting for between 70 and 90% of counts in a majority of cells, were used for dimensionality reductions (PCA and UMAP created on the first 30 PCs) and clustering; for each iteration of the pipeline, we chose the highest number of genes accounting for the target percentage of assigned counts. Seurat and Monocle were used for clustering with the same parameters as for the Smart-seq2 data (tables S6 and S7), with the adjustment that the kNN graph for Seurat used the five nearest neighbors. Imputation of expression levels was performed with ALRA (*86*); the imputed values were only used for UMAP visualization.

### Flow cytometry

MKs were stained with the corresponding antibodies (Table 1) in PBS/0.5% bovine serum albumin (BSA)/5 mM EDTA for 20 min at room temperature. They were then washed in PBS/0.5% BSA/5 mM EDTA, centrifuged for 8 min at 120*g*, and resuspended in PBS/0.5% BSA/5 mM EDTA with 1× 4′,6-diamidino-2-phenylindole (DAPI). They were analyzed using a Gallios flow cytometer (Beckman Coulter A94303) or sorted.

**Table 1.** Antibodies. FITC, fluorescein isothiocyanate; PE, phycoerythrin; HLA, human leukocyte antigen; IgG, immunoglobulin G.

**Antigen**	**Fluorophore**	**Supplier**	**Catalog number**	**Dilution**
MICB	Alexa 647	R&D Systems	FAB1599R-100UG	1:100
	APC	R&D Systems	FAB1599A-025	1:10
	PE	R&D Systems	FAB1599P-025	1:10
PVR	PE	MACS	130-105-905	1:10
	PECy7	BioLegend	337613	1:100
CD51	FITC	BioLegend	327908	1:100
CD168	Alexa 700	Novus Biologicals	NBP1-76538AF700	1:20
VEGFR1	Alexa 594	BioLegend	394903	1:20
CIP2A	APCCy7	Novus Biologicals	NB110-59722APCCY7	1:20
	APC	Novus Biologicals	NB110-59722APC	1:100
	FITC	Novus Biologicals	NB110-59722F	1:100
ANXA1	APC	BioLegend	831603	1:100
Vimentin	Alexa 488	BD Biosciences	562338	1:100
CD41	APCH7	BD Biosciences	561422	1:100
	APC	BD Biosciences	559777	1:10
	PE	BD Biosciences	555467	1:10
	FITC	BD Biosciences	555466	1:10
CD42	APC	Miltenyi Biotech	130-100-932	1:100
	FITC	BD Biosciences	558818	1:10
	BV605	BD Biosciences	743727	1:100
CD235	PE	BD Biosciences	555570	1:100
	PECy7	BD Biosciences	563666	1:100
	FITC	BD Biosciences	561017	1:10
CD71	PE	BD Biosciences	561938	1:100
	FITC	BD Biosciences	561939	1:100
HLA ABC	APC	Miltenyi Biotech	130-101-467	1:250
IgG control	APCCy7	BD Biosciences	560167	1:100
	APC	BD Biosciences	555751	1:10
	FITC	BD Biosciences	555748	1:10
	PE	BD Biosciences	555749	1:10
	PECy7	BD Biosciences	557872	1:100

**Table 2.** Putative progenitor markers. NK, natural killer cell; ERK1/2, extracellular signal–regulated kinase 1/2; DNAM-1, DNAX Accessory Molecule 1; BRCA1, Breast Cancer 1, Early Onset; MHC, major histocompatibility complex; MK, megakaryocyte; HSC, haematopoietic stem cell; CXCR4, C-X-C Motif Chemokine Receptor 4; MICB, MHC Class I Polypeptide-Related Sequence B; PVR, Poliovirus Receptor; ANXA1, Annexin A1; RHAMM, Receptor For Hyaluronan-Mediated Motility; VEGFR1, Vascular Endothelial Growth Factor Receptor 1.

**Marker**	**Function**
MICB	MHC class I–related protein (*54*) which binds to the NKG2D receptor on NKs or γδ T cells (*55*).
	Assembly is β2 microglobulin independent.
	Stress-associated marker (*54*), which can be secreted (*56*).
	Expression regulated by acetylation (*57*) of its heat shock element–containing promoter (*54*).
	Down-regulated in many cancers to evade the immune system (*58*).
PVR (CD155)	Transmembrane receptor that can also be secreted as a soluble factor (*59*). Involved in cell adhesion (*60*) via its role as a vitronectin receptor (*61*) as well as in the transendothelial migration of leukocytes (*62*).
	Binds to an NK receptor, DNAM-1 (*63*), which is expressed on platelets and is involved in MK and platelet adhesion to endothelial cells (*64*).
ANXA1	Induces HSC differentiation to granulocytes and macrophages (*65*).
CD168 (RHAMM)	Hyaluronic acid receptor (*66*) and also binds to mitotic spindle (*67*).
	Associates with BRCA1 and activates ERK1/2 downstream of CD44 (*67*).
	Expressed in actively cycling pre-HSCs from fetal liver (*68*).
	Cell cycle dependent and peaks in G_2_-M phase (*69*).
CD51	Expressed in fetal liver cells, which promote MK differentiation from HSCs (*70*).
	CD51^+^ perivascular cells promote platelet generation from MKs (*71*).
	CD41 expression replaces CD51 expression as HSCs differentiate (*72*).
VEGFR1	VEGF receptor expressed on monocytes, macrophages, HSCs, vascular smooth muscle cells, and leukemic cells (*73*).
	Increased expression in intermediates between HSCs and MKs (*74*).
	Activation leads to CXCR4 redistribution on MKs, movement toward vascular niche, and platelet production (*75*).

### Quantitative PCR

RNA was extracted using an RNeasy Mini Plus kit (Qiagen, 74104) with gDNA Eliminator columns. cDNA was synthesized using a Maxima First Strand cDNA Synthesis Kit (Thermo Fisher Scientific, K1641). qPCR was carried out using Applied Biosystems Fast or Power SYBR Master mix, with 0.5 μM primers (table S1) on an Applied Biosystems StepOne thermocycler. gDNA for qPCR was extracted using a Wizard SV kit (Promega, A2361) and analyzed as above.

### Selection of extended marker panel and index sorting

To identify markers that could further refine the MICB^+^PVR^+^ population, the 10x scRNA-seq data of the MICB^+^PVR^+^-sorted mature MKs were analyzed, and the top genes in each cluster were used for enrichment analysis (http://pantherdb.org). Clusters 1 and 2 had proliferation-associated GO terms (see main text). *ANXA1* was selected as a marker of cluster 1, and *HMMR* (CD168) was selected as a marker of cluster 2 (Fig. 3, G and H).

Next, the time-course Seurat object was filtered for D15 and D20 cells only. This was done to select only mature MKs to identify candidate markers that would work for MKPs in mature cultures and during differentiation. DE analysis was performed between clusters leading the mature MK cluster in trajectory analysis (clusters 6, 11, 15, and 34) (Fig. 1, G and J) and remaining clusters in the dataset. Genes that were differentially expressed between these target clusters and the rest of the dataset were visualized on the UMAP. The plots were manually curated to select candidate markers that were highly expressed in the clusters of interest and that could allow discrimination between clusters 6, 11, 15, and 34. Using the shortlist of differentially expressed genes, the following targets were selected, so that a combination of these four markers (fig. S4C) could discriminate between clusters: *ANXA1*, *HMMR* (CD168), *ITGAV* (CD51), and *FLT1* (VEGFR1); clusters 6 and 34: ANXA1^−^CD168^+^CD51^+^VEGFR1^−^; cluster 11: ANXA1^+^CD168^+^CD51^−^VEGFR1^−^; cluster 15: ANXA1^−^CD168^−^CD51^+^VEGFR1^+^.

Colony data from index sorts using these four markers could then allow us to ascertain which marker combination the MKPs expressed. This would then enable us to pinpoint the clusters that had the corresponding marker expression on both the progenitor and time-course objects. Together, this combined approach allowed us to select a minimal panel of surface markers that could select for MKPs.

We then look to validate these putative markers in functional assays using index sorts. Single cells were stained for these markers and index sorted (*87*) using a FACSAria II (BD Biosciences) or a FACSAria Fusion (BD Biosciences) into flat-bottomed, noncoated 96- or 384-well plates, which had been filled with 100 μl/30 μl of CellGenix GMP SCGM supplemented with SCF (25 ng/ml), TPO (20 ng/ml), and 1× PenStrep. The plates were incubated for 10 to 14 days at 37°C, 5% CO_2_, and 21% O_2_. The colonies were then counted and imaged.

### Bioinformatic methods for processing the flow cytometry output

Wells containing a colony of more than three MKs on D9 to D14 of culture were scored as positive observations; colonies containing fewer than three cells were scored as negative. To determine genes that were expressed differently in positive and negative observations, the Wilcoxon rank sum test was applied. The distribution of gene values and scatterplots of intensities of each gene pair were visualized, as well as PCAs of the data. Standard logistic regression and random forest models using 1000 decision trees were used to build classifiers for the observations; both sets of models failed to classify positive observations because of their low frequency [randomForest R package v4.6-14 (*80*)].

Across replicate plates, we observed considerable variability in the number of colonies. Two replicates, containing two and four colonies, respectively, were excluded from data augmentation and the bagging analysis because of their low number of colonies. Synthetic positive observations were generated using SMOTE (*88*) to create an augmented dataset with 1:1 ratio between positive and negative observations (no downsampling of negative observations was applied). The distributions of surface marker expressions in the augmented and original data were visualized and compared to assess changes arising from augmentation. Using the augmented data as training set, both standard logistic regression and RFs succeeded in classifying positive observations on the original dataset, at the cost of a higher false-positive rate for logistic regression.

To find the most discriminative features for separating positive and negative observations, the decision trees making up the random forest models were used. Specifically, the distribution of surface marker intensities used for splits and the ranks of a surface marker where it is first used in the tree were visualized. Surface markers with a consistently high rank (indicating that they are used early in trees to separate observations) and a tight distribution of intensities (indicating that the gene consistently separates observations at the same expression level) were considered good candidate markers. Separately, negative observations were subsampled to 30% of their original frequency to assess logistic regression, bagging decision trees, and random forest (the latter two using 1000 decision trees and otherwise default parameters) performance when trained on subsampled data; false-positive and false-negative rates were consistently higher than the corresponding model trained on augmented data, and subsampling was not used further.

To identify the most discriminative features for separating positive and negative observations/colonies, the decision trees making up the bagging models were used. Specifically, when traversing the trees depth first, the rank of the first occurrence of surface markers was recorded; subsequently, the distributions of ranks per surface marker and dataset were visualized using histograms and boxplots. Surface markers with consistently high rank across datasets were considered good candidates for improving the culture system. The rank distributions were summarized by calculating the means and medians of the ranks per surface marker and dataset, and the distributions of means and medians per surface marker were inspected.

### Methylcellulose colony-forming assay

Single cells were index sorted (*87*) using a FACSAria II (BD Biosciences) or a FACSAria Fusion (BD Biosciences) into round-bottomed BD Falcon tubes containing PBS/0.5% BSA/5 mM EDTA. The tubes were centrifuged at 120*g* for 8 min, and cells were resuspended in 300 μl of basal medium and added to 3.3 ml of nonenriched MethoCult (STEMCELL Technologies, H4535) supplemented with SCF (50 ng/ml; Gibco, PHC2116), thrombopoietin (100 ng/ml; R&D Systems, 288-TP), and 1× PenStrep (Gibco, 15140122). A total of 1.2 ml was plated in duplicate wells of a SmartDish (STEMCELL Technologies, 27370). Plates were incubated at 37°C, 5% CO_2_, and 21% O_2_, and colonies were counted after 8 to 14 days using an Olympus bright-field microscope.

## Notes

View/request a protocol for this paper from *Bio-protocol*.
