# Supplementary material for: Mapping the biogenesis of forward programmed megakaryocytes from induced pluripotent stem cells
Source: Sci Adv. 2022 Feb 16;8(7):eabj8618. doi: 10.1126/sciadv.abj8618 (PMC8849335; doi:10.1126/sciadv.abj8618)
Supplement: Supplementary file 1 — Figs. S1 to S6 Tables S1 and S9 [file sciadv.abj8618_sm.pdf]

Science Advances
AAAS

Supplementary Materials for

Mapping the biogenesis of forward programmed megakaryocytes from induced pluripotent stem cells

Moyra Lawrence, Arash Shahsavari, Susanne Bornelöv, Thomas Moreau, Rebecca McDonald, Thomas M. Vallance, Katarzyna Kania, Maike Paramor, James Baye, Marion Perrin, Maike Steindel, Paula Jimenez-Gomez, Christopher Penfold, Irina Mohorianu*, Cedric Ghevaert*

*Corresponding author. Email: cg348@cam.ac.uk (C.G.); iim22@cam.ac.uk (I.M.)

Published 16 February 2022, *Sci. Adv.* **8**, eabj8618 (2022)
DOI: 10.1126/sciadv.abj8618

The PDF file includes:

Figs. S1 to S6
Legends for tables S1 to S9
Tables S1 and S9

Other Supplementary Material for this manuscript includes the following:

Tables S2 to S8

Supplemental information titles and legends

Figure S1: Sorting megakaryocytes for CD34, KIT, CD61 or KDR does not enrich for MKPs. A. Flow cytometry plots of CD34, CD42, CD41, CD235 and KIT expression in a D45 A1ATD1 culture sorted for CD34 and KIT. Sorted cells were seeded in a methylcellulose colony formation assay, supplemented with SCF and TPO to promote MK formation. The resulting colonies were counted (central graph) and analysed by flow cytometry (pie charts), selecting viable cells by DAPI negativity and gating on isotype controls for each antibody. **B.** Flow cytometry strategy for sorting D29 A1ATD1 MKs for CD61 or KDR expression. Colony count from MKs sorted for the markers shown and plated in a methylcellulose colony formation assay, supplemented with SCF and TPO. Colonies were scored on D15. **C.** Transgene expression strategy for 10x Genomics sequencing. V(D)J primers from the target enrichment library preparation protocol were replaced with primers for regions in the lentiviral backbone, allowing libraries to be generated of the overlap regions between transcript and lentiviral backbone, ultimately providing a readout of transgene expression levels**.** LTR: Long Terminal Repeat. EF1α: Elongation Factor 1α promoter, WPRE: woodchuck hepatitis virus posttranscriptional regulatory element. Primer sequences in Supplementary Table 1.

Figure S2: Time-course quality control summaries suggest that biological variation and not technical effects are the primary driver of post-normalisation transcriptional heterogeneity. A-D. Distributions of number of features (A), unique molecular identifiers (UMI) (B), mitochondrial (MT) reads % (C), and ribosomal protein (RP) reads % (D) across cells, split per sample illustrate differences across the time points. **E.** Scatter plot for evaluating sequencing saturation. The logarithmic relationship observed for UMI counts versus number of detected features per cell suggest that sequencing saturation was not reached. **F.** Histogram illustrating the number of captured cells per sample (target cells per time point: 2000). The observed variation underlines the heterogeneity in resolution across the time-course; note the y-axis is on a log10 scale. **G.** Batch summary across the time points. UMAP of cells coloured by biological batch illustrates some overlap across the two batches, suggesting that the dimensionality reduction is not driven primarily by biological batch. Inset panel: D5 samples across batches. **H.** Sequencing run summary across the time points. UMAP of cells coloured by sequencing run illustrates an acceptable overlap and suggests no significant batch effect. **I-J.** UMAPs coloured by raw (I) and normalized (J) sequencing depth illustrate that normalization reduces differences across UMAP regions. **K-L.** UMAPs presenting the MT% (K) and RP% (L) across cells using a colour gradient illustrate regions of UMAP space high in MT% or RP%; these regions are, in their majority, disjunct. **M.** UMAP faceted by cell origin, coloured by Monocle cluster. Samples corresponding to the same timepoint but different sequencing run or biological batch are similar both in location in UMAP space and in cluster membership. Example: D5.2: Day 5, batch 2.

Figure S3: Identification of cell identity clusters on the UMAP embedding of the single cell RNA sequencing of megakaryocyte differentiation. A. 51 Monocle clusters shown on the UMAP of the MK differentiation process. **B and C.** Normalised expression levels of transcripts associated with the naïve (B) and primed (C) pluripotent state in the monocle clusters shown in Figure S3A corresponding to iPSCs. **D.** sctransform-normalised *HAND1*, *KRT8*, *KRT18*, *KRT19*, *CD34*, *KDR*, *CDH5*, *PECAM*, *GATA1*, *TAL1*,

FLI1, *GYPA*, *ITGA2B*, *GP1BA*, *CD59*, *THY1*, *CD38* and *KIT* expression shown on the UMAP of the MK differentiation time-course. **E.** Random forest-predicted cell types on single cell time-course data. Cell types learned on 10x Genomics haematopoietic stem and progenitor cells isolated directly from human bone marrow, spleen and peripheral blood[57]; model deployed here trained on donor 2: 3% D0 cutoff, threshold of 0.62. Non-overlapping groups of MEP and MkP are observed during *in vitro* MK differentiation. MEP: Megakaryocyte Erythroid Progenitor, MkP: Megakaryocyte Progenitor, Donor 2: 1% D0 cutoff, threshold of 0.667.

Figure S4: Expression of putative megakaryocyte progenitor markers during megakaryocyte differentiation A. Heatmap of most significant temporally changing genes defining the monocle trajectory shown in Figure 1J. Each row shows the expression of one gene on a random subset of cells across the pseudotime trajectory. **B.** Unnormalised *GATA1*, *TAL1* and *FL11* expression shown on the UMAP of the scRNA-seq experiment of the MK forward programming time-course in enriched libraries and gene expression library. **C.** Normalised *MICB*, *PVR*, *ANXA1*, *HMMR*, *ITGAV* and *FLT1* expression shown on the time-course UMAP (Figure 1F). **D.** Flow cytometry analysis of PVR and MICB expression in A1ATD1 MKs. Cells were stained with PVR or MICB antibodies or corresponding isotype control (when available) and analysed using a Gallios flow cytometer. Positive cells were gated on the isotype control (PVR_upper panels) or unstained (MICB_lower panels) samples. In the upper panel, the purple region of the histogram represent cells that are PVR positive (compared to isotype). In the lower panel, the dark blue histogram represent the whole population of which 74% were MICB positive vs the unstained control. This was further sub-divided according to levels of PVR expression (see Figure 2G): light blue (PVR low), dark purple (PVR medium) and green (PVR high).

Figure S5: Quality control summaries of Smart-seq data and MKP markers in human peripheral blood HSC-derived MK cultures. A. The distribution of unnormalized sequencing depths (ncount) show high variation across cells; cells with ncount > 200,000 are further investigated. **B.** Distribution of normalised (using sctransform) sequencing depths per cell post-filtering; a tighter distribution of normalised ncounts is obtained; no further filtering is applied. **C-D.** Scatterplots of sequencing depth versus number of detected features per cell that indicate sequencing saturation; values on the x-axes are on linear (C) and log2 (D) scale; values on the y-axes are on linear scale. **E.** Boxplots of bagging analysis, performed on repeatedly subsampled data from 20 index sorted A1ATD1 MKs stained for the markers shown on the x axis. **F.** Colony count from CFU assays on human peripheral blood-derived MKs. $CD34^+$ cells were isolated from the leukocyte depletion cones of three healthy human adult donors and differentiated into MKs. On D5, MKs were sorted as either live or live and $MICB^{hi}PVR^{hi}CD51^{hi}CD168^{hi}VEGFR1^{lo}$ using the same gating strategy as for iPSC-derived MKs and plated in CFU assays in technical duplicate. Colonies were counted 8 days later and are shown as the mean of two technical replicates per biological triplicate. ***** $0.05 < p < 0.01$ Adjusted p value compared to Live cells by two-tailed t-test.

Figure S6: MKP sequencing quality control summaries and the optimisation of MKP generation by cytokine and oxygen titration A. Distributions of unique

molecular identifiers (UMIs), number of detected features, % of reads incident to mitochondrial genes (MT) and ribosomal proteins (RP) illustrate variability among cells prior to filtering and normalization; cells with nfeature < 1,000 or MT% > 10% were discarded from downstream analysis. **B.** Scatter plot for evaluating sequencing saturation. The logarithmic relationship observed between the number of detected features versus UMI counts per cell suggests sequencing saturation has not been achieved. **C.** Distributions of UMI counts, number of features, MT% and RP% post filtering and normalisation. The tighter distributions suggest an increased comparability of the cells. **D-E.** UMAP embeddings presenting the MT% (D) and RP% (E) across cells using a colour gradient illustrate regions of UMAP space high in MT% or RP%; these regions are, in their majority, disjunct. **F.** Unnormalised *GATA1*, *TAL1* and *FL11* expression in enriched libraries and gene expression library shown on the UMAP embedding of the scRNA-seq of sorted $MICB^{+}PVR^{+}$ MKPs. Two enriched libraries were sequenced for each transgene combination. **G.** Change in cell number, progenitor number and mature MK number on D15-D26 of QOLG1 MK differentiation. Data shown in Figure 6 with each timepoint normalised to the previous timepoint.

Supplementary Table 1: Sequences of primers used for qPCR and 10x Genomics enriched library amplification.

Supplementary Table 2: Samples used for single cell RNA sequencing of *in vitro* megakaryocyte differentiation time-course. Statistics are shown pre- and post-filtering.

Supplementary Table 3: Markers of each monocle cluster from time-course single cell RNA sequencing experiment.

Supplementary Table 4: GSEA terms associated with each monocle cluster from time-course single cell RNA sequencing experiment.

Supplementary Table 5: Cells identified by random forest model as corresponding to *in vivo* haematopoietic intermediates. Numbers of cells from each timepoint which were classed as each cell type from both donors. EryP: erythroid progenitor, HSC/MPP: haematopoietic stem cell/multipotent progenitor, MEP: megakaryocyte erythroid progenitor, MkP: megakaryocyte progenitor.

Supplementary Table 6: GO terms associated with each monocle cluster from $MICB^{+}PVR^{+}$ MKP single cell RNA sequencing experiment.

Supplementary Table 7: Markers of each monocle cluster from $MICB^{+}PVR^{+}$ MKP single cell RNA sequencing experiment.

Supplementary Table 8: GSEA terms associated with each monocle cluster from $MICB^{+}PVR^{+}$ MKP single cell RNA sequencing experiment.

Supplementary Table 9: Sensitivity and Specificity table for markers. Index sort data from two independent sorts of A1ATD1 MKs was analysed for the sensitivity and specificity of the cutoffs selected for MKP enrichment (Figure 4b). First dataset was

filtered on PVR (top 20%) and MICB (top 50%), then CD51 (top 85%) and CD168 (top 62%). Second dataset was filtered on PVR (top 20%) expression only. Specificity is defined as non-colonies excluded by the markers over total non-colonies. Sensitivity is defined as colonies selected by the markers over total colonies.

Figure S1

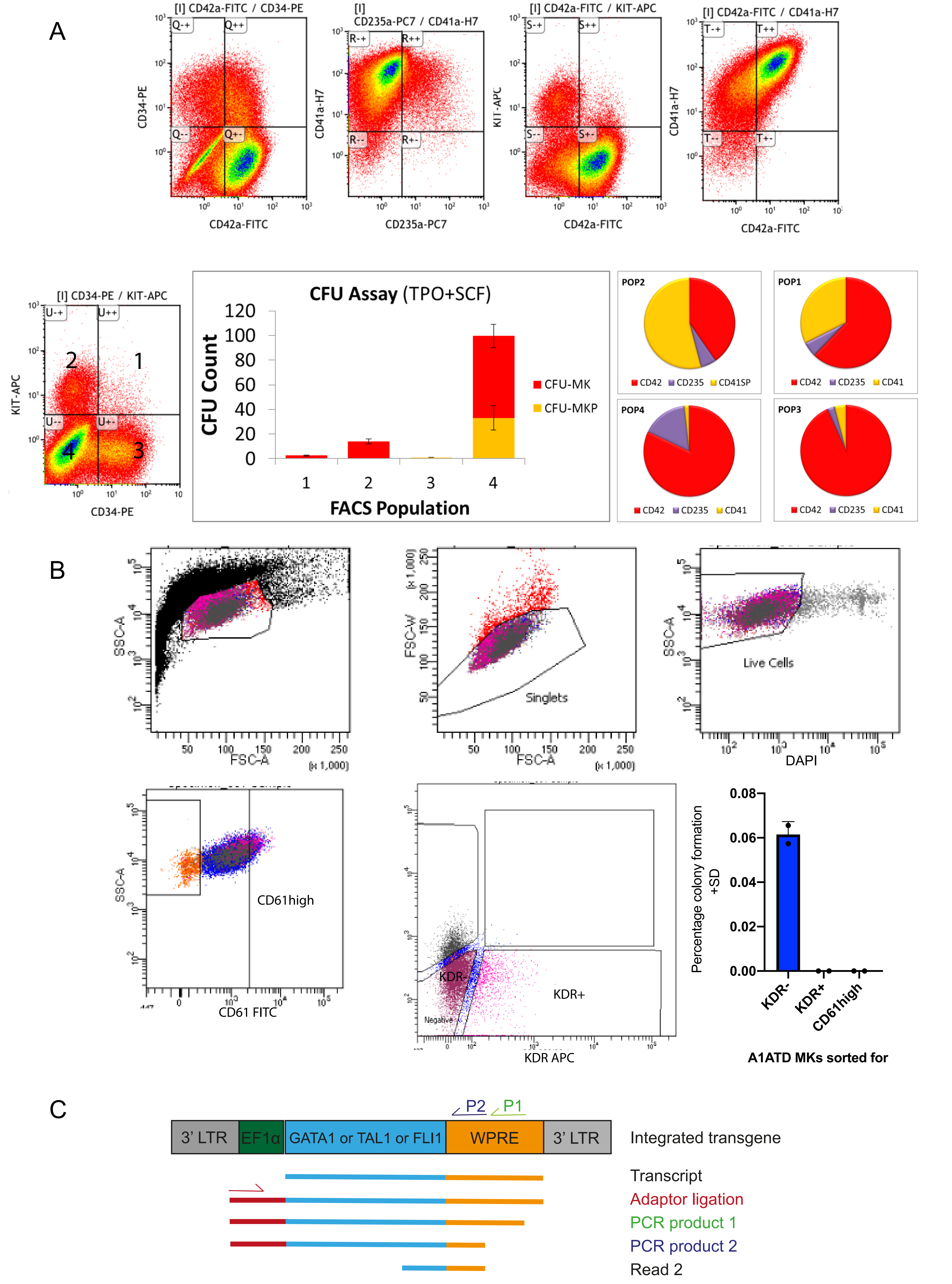
A
[I] CD42a-FITC / CD34-PE
CD34-PE
CD42a-FITC
[I] CD235a-PC7 / CD41a-H7
CD41a-H7
CD235a-PC7
[I] CD42a-FITC / KIT-APC
KIT-APC
CD42a-FITC
[I] CD42a-FITC / CD41a-H7
CD41a-H7
CD42a-FITC
[I] CD34-PE / KIT-APC
KIT-APC
CD34-PE
CFU Assay (TPO+SCF)
CFU Count
FACS Population
CFU-MK
CFU-MKP
POP2
CD42 CD235 CD41SP
POP1
CD42 CD235 CD41
POP4
CD42 CD235 CD41
POP3
CD42 CD235 CD41
B
SSC-A
FSC-A
FSC-W
Singlets
Live Cells
DAPI
CD61high
CD61 FITC
KDR-
KDR+
Negative
KDR APC
Percentage colony formation +SD
KDR-
KDR+
CD61high
A1ATD MKs sorted for
C
P2
P1
3' LTR
EF1α
GATA1 or TAL1 or FLI1
WPRE
3' LTR
Integrated transgene
Transcript
Adaptor ligation
PCR product 1
PCR product 2
Read 2

Figure S2

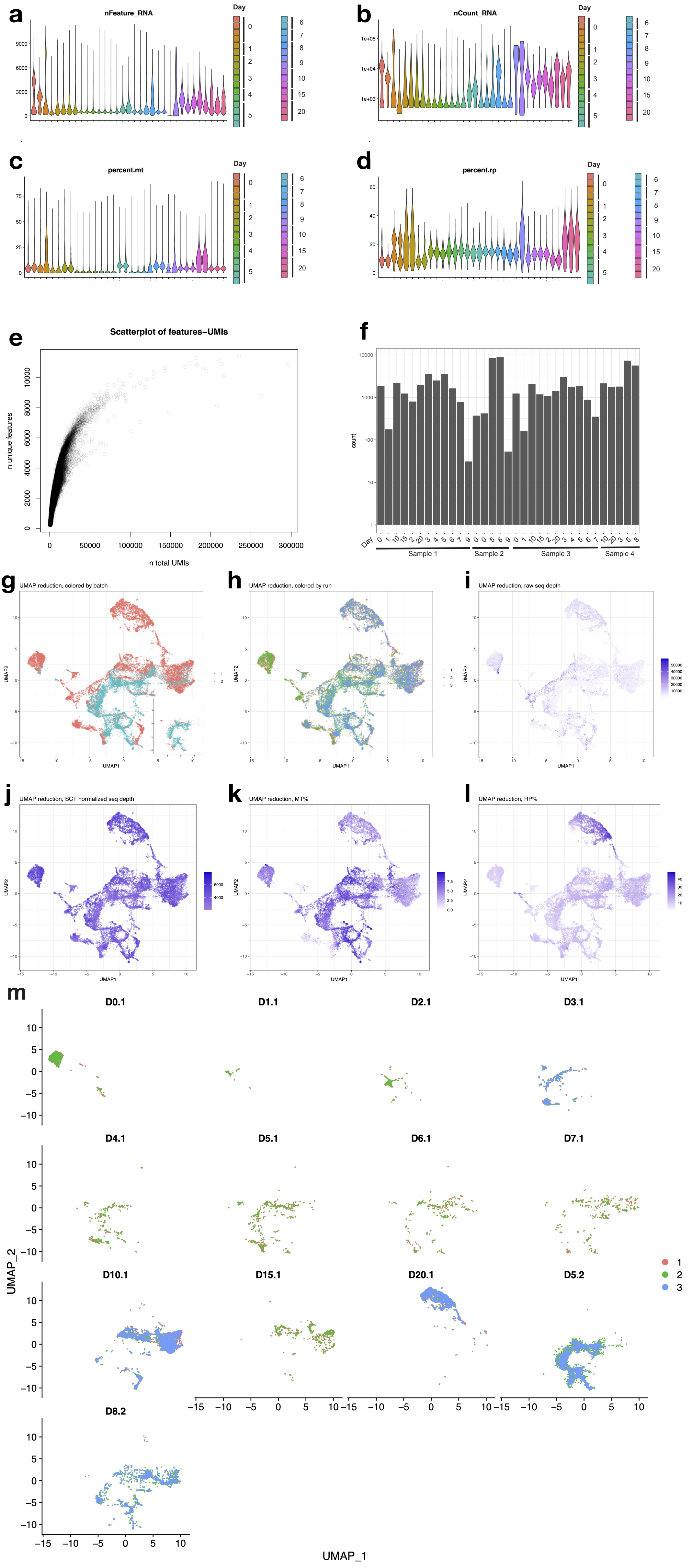
a
nFeature_RNA
Day
0
1
2
3
4
5
6
7
8
9
10
15
20
b
nCount_RNA
c
percent.mt
d
percent.rp
e
Scatterplot of features–UMIs
n unique features
n total UMIs
f
count
Day
Sample 1
Sample 2
Sample 3
Sample 4
g
UMAP reduction, colored by batch
h
UMAP reduction, colored by run
i
UMAP reduction, raw seq depth
j
UMAP reduction, SCT normalized seq depth
k
UMAP reduction, MT%
l
UMAP reduction, RP%
UMAP1
UMAP2
m
D0.1
D1.1
D2.1
D3.1
D4.1
D5.1
D6.1
D7.1
D10.1
D15.1
D20.1
D5.2
D8.2
UMAP_1
UMAP_2

Figure S3

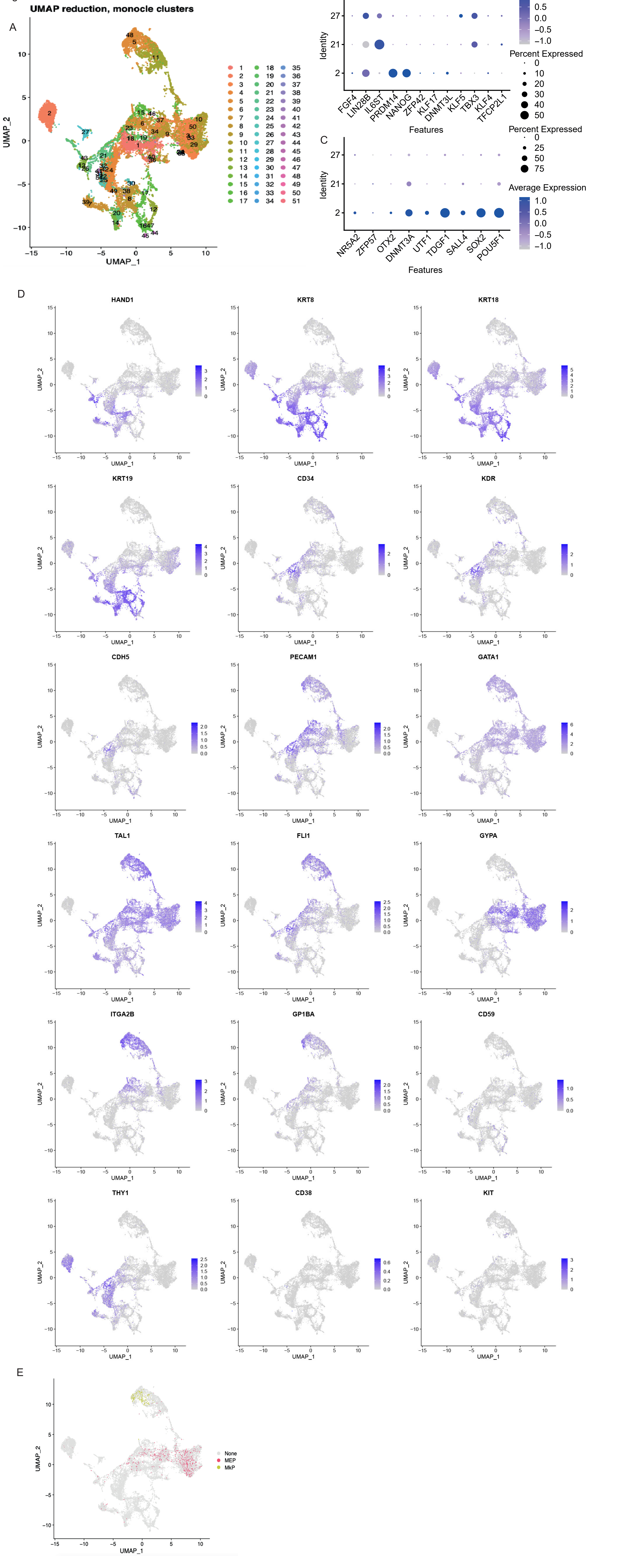

A
UMAP reduction, monocle clusters
UMAP_1
UMAP_2
B
Average Expression
Percent Expressed
Identity
FGF4
LIN28B
IL6ST
PRDM14
NANOG
ZFP42
KLF17
DNMT3L
KLF5
TBX3
KLF4
TFCP2L1
Features
C
Percent Expressed
Average Expression
Identity
NR5A2
ZFP57
OTX2
DNMT3A
UTF1
TDGF1
SALL4
SOX2
POU5F1
Features
D
HAND1
KRT8
KRT18
KRT19
CD34
KDR
CDH5
PECAM1
GATA1
TAL1
FLI1
GYPA
ITGA2B
GP1BA
CD59
THY1
CD38
KIT
E
None
MEP
MkP

Supplementary Figure 4

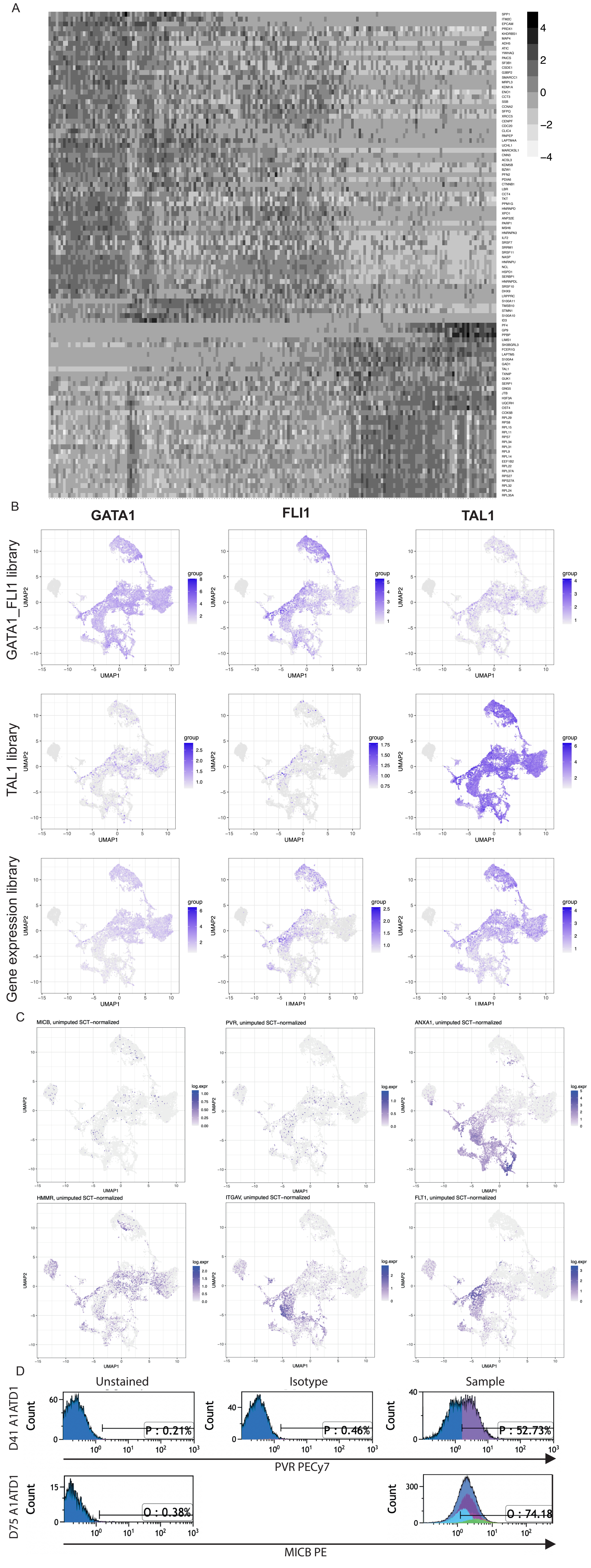

Figure S5

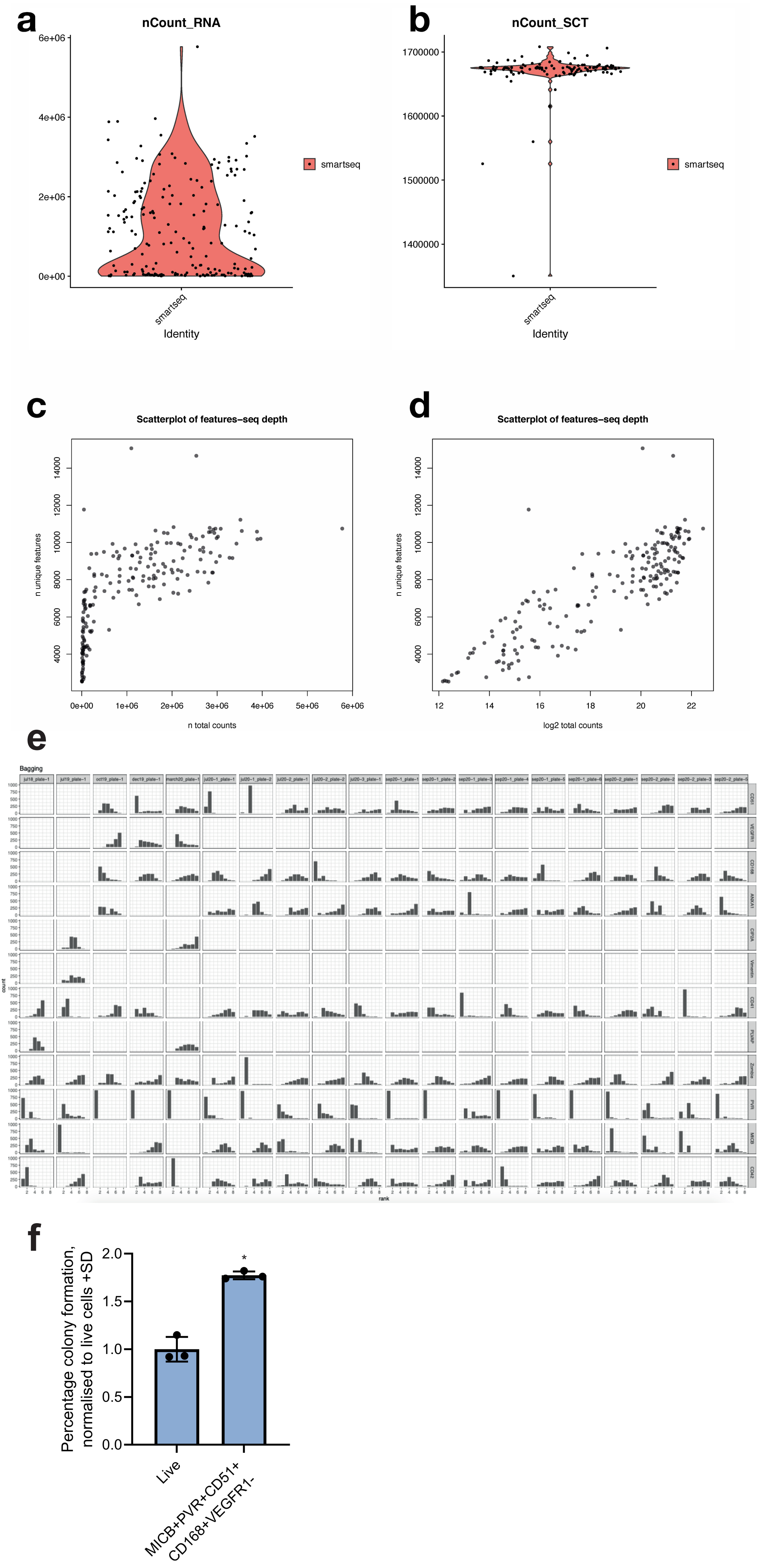
a
nCount_RNA
6e+06
4e+06
2e+06
0e+00
smartseq
Identity
b
nCount_SCT
1700000
1600000
1500000
1400000
smartseq
Identity
c
Scatterplot of features–seq depth
n unique features
n total counts
d
Scatterplot of features–seq depth
n unique features
log2 total counts
e
Bagging
count
rank
f
Percentage colony formation, normalised to live cells +SD
2.0
1.5
1.0
0.5
0.0
*
Live
MICB+PVR+CD51+ CD168+VEGFR1-

Figure S6

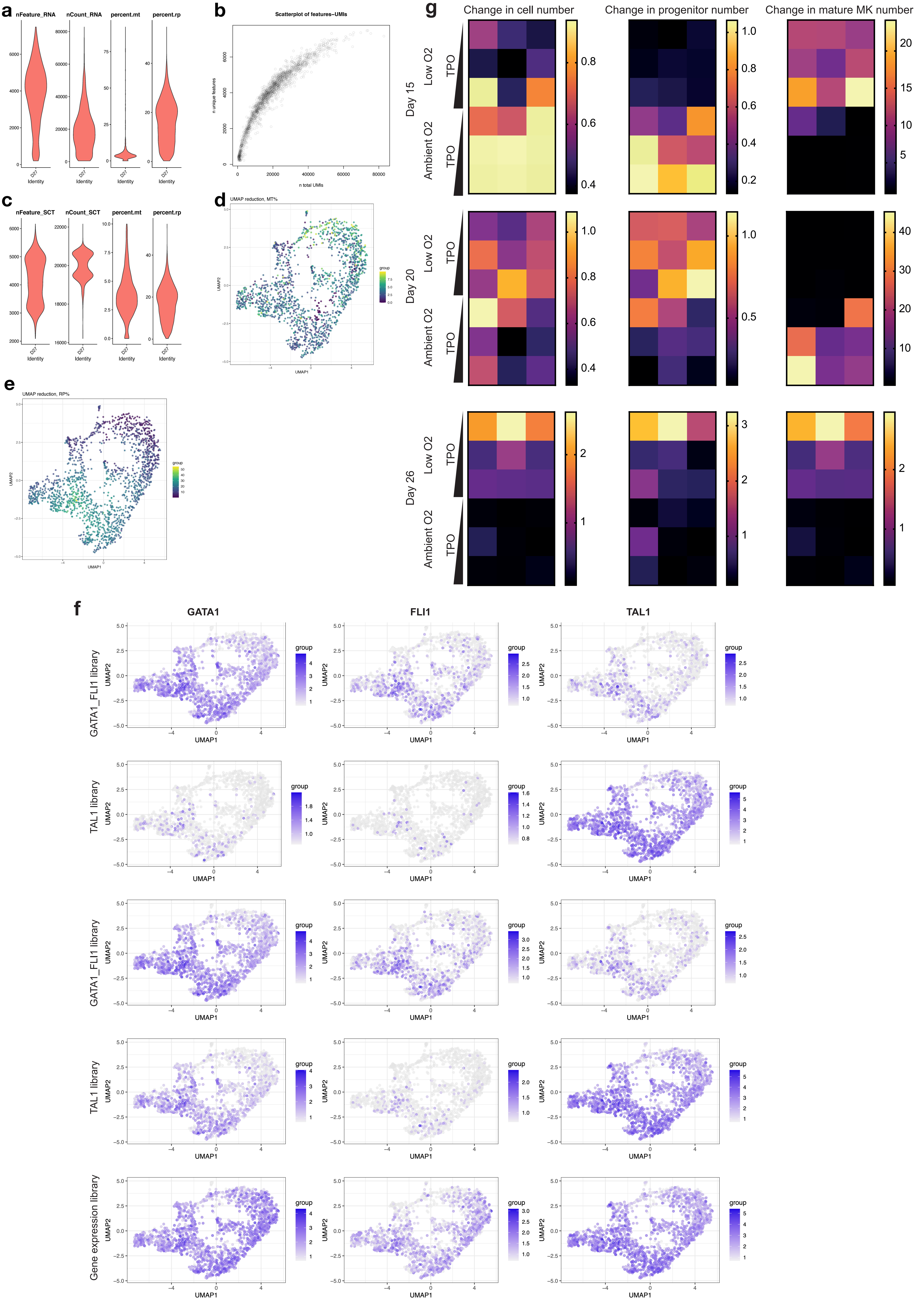
a
nFeature_RNA
nCount_RNA
percent.mt
percent.rp
D27
Identity
b
Scatterplot of features–UMIs
n unique features
n total UMIs
c
nFeature_SCT
nCount_SCT
percent.mt
percent.rp
d
UMAP reduction, MT%
e
UMAP reduction, RP%
UMAP1
UMAP2
group
g
Change in cell number
Change in progenitor number
Change in mature MK number
Day 15
Day 20
Day 26
Low O2
Ambient O2
TPO
f
GATA1
FLI1
TAL1
GATA1_FLI1 library
TAL1 library
Gene expression library

Supplementary Table 1: Primer sequences

qPCR primers

Target	Forward primer	Reverse primer
ITGA2B	TTCCTACTACCAGAGGCTGCATCGG	GGAGCGCCCACCAGCAGATCAT
GP1bA	GCCTTACACTCGCCTCACTC	TTGTGGGATAGATCCAGGGTC
MYB	TGCCTGGACGAACTGATAAT	GCAGGGAGTTGAGCTGTAGG
MICB	GCAGAGGTTCACCTGCTACA	TGGAAAGTCTGTCCGTTGACT
BRCA1	AAGCAGCGGATACAACCTCA	TTCTTGATCTCCCACACTGCAA
CIP2A	GGAGTGGTTTGTCGGAGCAG	GGCACCAGAATAGAAAATTTTGACA
FEN1	CGAACCAAGCTTTAGCCGC	AGTTTGGCCAGGCCTTGAAT
FANCG	AGCCTCACCCCTTCATTGTG	GGCCAGCAGGTCCAAGTAAT
ORC1	GCAGCAGATCCTAAGGTCCC	GTGCATCTCCAGACAGTGCT
KNTC1	GGGTACCTGAGTGTCGGTTC	GCTGCATGCCTGTATCTTTGG
PPIAb	GTTCTTCGACATTGCCGTCG	TCTGTGAAAGCAGGAACCCT
HMBS	GGGAACCAGCTCCCTGCGAAG	AGCTGTTGCCAGGATGATGGCAC
GAPDH	AAATCAAGTGGGGCGATGCT	CAAATGAGCCCCAGCCTTCT
RPL15	GCGCCGACTGGGCTACAAGG	ACTGGGCGTTTTCGGCCACC
Beta globin	GCTTCTGACACAACTGTGTTCACTAGC	CACCAACTTCATCCACGTTCACC
Telomeric repeats	CGGTTTGTTTGGGTTTGGGTTTGGGTTTGGGTTTGGGTT	GGCTTGCCTTACCCTTACCCTTACCCTTACCCTTACCCT

10X amplification primers

Target	Sequence
GATA1 and FLI1 transgene inner	CCTCTGGATTACAAAATTTGTGAAAG
	GCAGCGTATCCACATAGCGT
	GCCATACGGGAAGCAATAGCA
GATA1 and FLI1 transgene outer	CACTGTGTTTGCTGACGCAAC
	TGCACACCACGCCAC
	GTGCACACCACGCCA
TAL1 transgene inner	CCTAGAAAAACATGGAGCAATC
	ATCCCAGCAGCCTAAGAACG
	AAGTTCAAGTCCACCGCCTT
TAL1 transgene outer	GGGCTAATTCACTCCCAACGA
	AGGCACAATCAGCATTGGTA
	TGTGACTGGAAAACCCACC

Supplementary Table 9: Sensitivity and Specificity table for markers. Index sort data from two independent sorts of A1ATD1 MKs was analysed for the sensitivity and specificity of the cutoffs selected for MKP enrichment (Figure 4b). First dataset was filtered on PVR (top 20%) and MICB (top 50%), then CD51 (top 85%) and CD168 (top 62%). Second dataset was filtered on PVR (top 20%) expression only. Specificity is defined as non-colonies excluded by the markers over total non-colonies. Sensitivity is defined as colonies selected by the markers over total colonies.

**Marker**	**Specificity**	**Sensitivity**
PVR	0.7996109	0.6111111
MICB	0.4931907	0.7222222
CD51	0.1390728	0.7777778
CD168	0.384106	0.7777778
VEGFR1	0.7439024	0.2
**After filtering for PVR**		
CD51	0.1624549	0.972973
CD168	0.3465704	0.7027027
VEGFR1	0.7515528	0.32
